# Does Children’s Education Improve Parental Health and Longevity? Causal Evidence from Great Britain

**DOI:** 10.1177/00221465221143089

**Published:** 2023-01-27

**Authors:** Cecilia Potente, Patrick Präg, Christiaan W. S. Monden

**Affiliations:** 1 University of Zurich, Zurich, Switzerland; 2 CREST, ENSAE, Institut Polytechnique de Paris, France; 3 University of Oxford, Oxford, UK

**Keywords:** causal inference, education, health, intergenerational relationships, mortality

## Abstract

Parents with better-educated children are healthier and live longer, but whether there is a causal effect of children’s education on their parents’ health and longevity is unclear. First, we demonstrate an association between adults’ offspring education and parental mortality in the 1958 British birth cohort study, which remains substantial—about two additional years of life—even when comparing parents with similar socioeconomic status. Second, we use the 1972 educational reform in England and Wales, which increased the minimum school leaving age from 15 to 16 years, to identify the presence of a causal effect of children’s education on parental health and longevity using census-linked data from the Office for National Statistics Longitudinal Study. Results reveal that children’s education has no causal effects on a wide range of parental mortality and health outcomes. We interpret these findings discussing the role of universal health care and education for socioeconomic inequality in Great Britain.

Those with better education enjoy better health (Galama, Lleras-Muney, and van Kippersluis 2018), and the benefits of education may also spill over to one’s peers’ health, such as spouses (Huijts, Monden, and Kraaykamp 2010) and siblings (Kravdal 2008). Recently, intergenerational health benefits of education have received increasing attention because better-educated parents have healthier children (Vollmer et al. 2017) and parents to better-educated children live longer and healthier (Elo, Martikainen, and Aaltonen 2018; Thoma et al. 2021; Wolfe et al. 2018a, 2018b). However, the spillovers from children to parents are understudied (De Neve and Kawachi 2017), especially in terms of the causal relationship (Hu and Bobak 2018). In this study, we ask two questions:

*Research Question 1:* Are adult children’s educational attainment and parental health and mortality associated in Great Britain?
*Research Question 2:* Does an increase in compulsory education produce benefits for population health through spillover effects from adult children to their parents?

Life course theory has long emphasized the importance of linked lives, the fact that life courses of significant others are interlocked (Elder, Johnson, and Crosnoe 2003). In line with this reasoning, children’s life courses—including their educational attainment—can be expected to have implications for their parents’ lives. We connect this theoretical perspective with “fundamental cause” theory (Clouston and Link 2021), which stresses the role of individuals leveraging resources to gain health advantages in the emergence of health inequalities. Based on this, our study examines how far parents are able to utilize the resources of significant others—namely, the education of their children—to improve their own health.

Children’s education is correlated with a number of different parental health outcomes: Parents of better-educated children are less depressed (Lee et al. 2017; Sabater and Graham 2016b; Wang et al. 2021; Wu and Penning 2019; Yahirun, Sheehan, and Mossakowski 2020), have better cognitive functioning (Ma et al. 2021; Yahirun, Vasireddy, and Hayward 2020), suffer less from functional limitations (Yahirun, Sheehan, and Hayward 2017; Zimmer, Hermalin, and Lin 2002) and inflammation (Lee 2018), and live longer (Elo et al. 2018; Friedman and Mare 2014; Jiang 2019; Sabater and Graham 2016a; Torssander 2013, 2014; Wolfe et al. 2018a, 2018b; Yahirun et al. 2017; Yang, Martikainen, and Silventoinen 2016). This has become known as the “social foreground” hypothesis (Torssander 2013). The evidence so far suggests there might be health benefits to children’s education that extend beyond their own lives and that investing in education is an effective strategy for improving the living conditions of a wider range of people. Some of the reported health benefits are large; Friedman and Mare (2014), for instance, report that having a child with college education rather than a child who has not finished high school goes along with almost two additional years of life in the United States. However, unobserved heterogeneity poses serious threats to the validity of the causal link. Because life expectancy and the relationship between offspring and mother/father differ by gender, we examine men and women separately in all our analyses.

Causality is the crucial issue for the analysis of educational spillover effects. The association between children’s education and parental longevity might be driven by endowments of the parental family, either socioeconomic or genetic, that affect both children’s education and parental longevity. However, across existing studies, the approaches used to rule out confounders often have considerable limitations. While Torssander (2013) is able to include sibling fixed effects in the parental generation that control for family background factors, all other associational studies are rarely able to include comprehensive controls for parental socioeconomic status (SES). Friedman and Mare (2014), for instance, control for parental income, wealth, and both parents’ education, but many studies have only a few control variables for the SES of the older generation at their disposal (e.g., only occupation [Zimmer, Hanson, and Smith 2016] or education [Zimmer et al. 2007]). At the same time, however, the question remains to what extent these SES indicators are actually confounders and not pathway variables by which children’s education exerts its positive effects and whether accounting for them biases the findings for children’s education (De Neve and Kawachi 2017). A confounding pathway that would link offspring education to parental longevity could be a genetic one, and indeed, genetic variants predicting both education and longevity have recently been identified (Krapohl et al. 2014; Marioni et al. 2016).

A natural experiment allows overcoming the problems of confounding, observed or unobserved. Quasi-natural experiments exploit the variation in the key explanatory variable—in this case, education—that is caused by factors outside individuals’ power, such as a policy change. Our study uses the 1972 educational reform in England and Wales, which increased the minimum school leaving age from 15 to 16 years, to identify the presence of a causal effect of children’s education on parental health and longevity by means of a regression discontinuity design. The intuition is that we compare children born just before the date after which they have to stay a year longer in school to those children who were born just after the cutoff date. The assumption is that the negligible difference in birth timing is quasi-random and that the two groups of children differ only in whether they receive the treatment (being required to stay one year longer in school) or not and do not differ in other observed or unobserved characteristics.

The one-year increase in compulsory schooling is a good opportunity to explore the causal link between adult offspring’s education and health for three reasons. First, the 1972 reform has affected a large proportion of the cohort in question, around 25% (Clark and Royer 2013), which is much higher compared to reforms in other countries, such as the United States, Norway, Canada, and France (Albouy and Lequien 2009; Black, Devereux, and Salvanes 2008; Lleras-Muney 2005; Oreopoulos 2006). Second, the 1972 education reform had important consequences for the affected cohorts. Research has demonstrated large effects on wages and labor market outcomes (Devereux and Hart 2010; Grenet 2013; Oreopoulos 2006) and some health benefits (Davies et al. 2018). Although an extra year of schooling might seem a modest amount to identify educational differences, increasing the age at school leaving for an entire cohort represents a major effort for policymakers and educational practitioners. The 1944 Educational Act raised the minimum school-leaving age from 14 to 15 years in 1947 and put provisions into place for a second one-year increment that was to follow once conditions allowed. It took another 25 years until this happened, emphasizing the size of the undertaking that such a one-year increment means in practice. Third, the reform restricted itself to increasing the mandatory school leaving age without implementing other changes in the educational system, making it easier to identify an effect of the reform.

Educational spillover effects have important policy implications. Improving the education of younger generations could prove to be a useful policy instrument in contexts where older populations are growing without precedence (Lutz, Sanderson, and Scherbov 2008) because it might serve as a public health intervention that affects not just younger but also older generations. Policies creating such intergenerational spillovers would also alleviate concerns about generational fairness of policy measures, in the sense that other interventions for the benefit of one generation come at the expense of another generation (Friedman and Mare 2014). If education is a family resource that provides benefits across generational boundaries, investing in education is not a zero-sum game from a generational perspective, and a focus on the individual returns to education would underestimate the societal returns overall.

We contribute to the study of “social foreground” in four ways. Our first contribution is to document the association between adult children’s education and parental mortality in Great Britain, a context with limited public welfare provision yet a universal health care system, for the first time. We use high-quality birth cohort data to document the association. Our second contribution is to examine the causal nature of the association between children’s education and parental health with linked census data from England and Wales, whose large sample size allows for precise estimates. We further make two minor contributions. Third, we employ a regression discontinuity approach to identify the causal effect of children’s education on parental mortality and health. Regression discontinuity approaches have an advantage over the instrumental variable approaches used in the existing literature because the regression discontinuity approach allows to better discern the causal impact of the reform from a secular time trend in the data (Galama et al. 2018). Our fourth minor contribution is to extend our analysis beyond mortality and include self-reported health and long-standing illness as outcomes. Some mixed results found in the literature so far could be because of the wide range of outcomes being analyzed separately. By reporting results for a large number of different outcomes, we aim to obtain a comprehensive picture of the causal effect of offspring’s education on parental health.

Our article proceeds as follows. In the next section, we review the existing literature and identify mechanisms linking adult children’s education and parental health. In a first empirical analysis, we use high-quality birth cohort data of individuals born all in one week of 1958 (Power and Elliott 2006) to describe the association between adult children’s education and parental longevity. This part of the analysis is to show that there is a clear relationship between changes in children’s schooling and parental mortality in those cohorts. In the second empirical analysis, we exploit an educational reform affecting the birth cohorts from 1957 onward and linked census data (Shelton et al. 2019) to assess whether the association between adult children’s education and parental health can be considered causal. These two analyses exploit individual strengths of the two data sets and complement each other. The 1958 cohort study can be used to show the association between educational attainment and parental life expectancy, yet it does not allow ruling out crucial confounding factors using a quasi-experimental design. The census data allow us to use a quasi-experimental design because children vary in their birth dates around the time of an educational reform but do not provide information on children’s educational attainment necessary to establish association.^1^ We conclude by discussing implications for universal health care and the role of education in socioeconomic inequality in Great Britain.

## Background

### What We Know So Far

Only few studies use exogenous variation to identify a causal effect of adult children’s education on parental health (De Neve and Fink 2018; Ludwig et al. 2021; Lundborg and Majlesi 2018; Ma 2019; Ma et al. 2021), relying on schooling reforms to instrument the parental health effects of an increase in children’s education. Most studies are based in low- and middle-income countries (De Neve and Fink 2018; Ludwig et al. 2021; Ma 2019; Ma et al. 2021), showing, for example, a 30% increase (1.1 years) in children’s education reduced parental mortality in Tanzania by 3.7 percentage points for mothers and .8 percentage points for fathers (De Neve and Fink 2018). The only study in a resource-rich setting, Sweden, does not find any overall effect (Lundborg and Majlesi 2018). Only daughters’ education has positive effects for fathers, particularly low-educated fathers, and almost a third of the increase in the likelihood to live to age 80 between 1943 and 1955 is due to an increase in daughters’ schooling. However, the compulsory schooling reforms in Sweden did not only increase the number of years of schooling but also brought about other changes in the educational system, introducing a new curriculum as well as tracking, raising the question as to what exactly of the reform caused the improvement in fathers’ health. Moreover, the few studies that used admission lotteries for health care professionals in Sweden and the Netherlands show mixed evidence for children’s effects on parental health (Artmann, Oosterbeek, and van der Klaauw 2022; Chen, Persson, and Polyakova 2022).

### Theoretical Mechanisms

There are various hypotheses on how adult children’s education could be linked to parental health. First, better-educated children can induce better health behaviors in their parents, which lead to greater longevity. This would be consistent with the association found by Friedman and Mare (2014). They show that the association between children’s education and parental longevity is particularly strong when it comes to lung cancer and chronic respiratory diseases, two causes of death linked to smoking. However, Ma (2019) does not find any causal effect of offspring’s education on parents’ smoking and drinking intensity in China. In Great Britain, risky health behaviors, such as alcohol consumption and smoking, have been highly prevalent across the twentieth century and decreased only in recent years. Prevalence of binge drinking and obesity in Great Britain are still above the European average (OECD and European Observatory on Health Systems and Policies 2017). Compared to the United States, Great Britain has similar smoking patterns but lower obesity and greater heavy drinking prevalence (Banks et al. 2006). In general, old-age health for U.S. residents appears worse than for British people across the whole socioeconomic distribution (Banks et al. 2006; Banks, Muriel, and Smith 2010).

Second, better-educated children are able to generate higher earnings (Devereux and Hart 2010; Grenet 2013; Oreopoulos 2006), which would allow them to financially support their parents. For instance, De Neve and Fink (2018) interpret their finding that sons’ education is more beneficial for parental, particularly maternal, health in Tanzania as being due to greater labor market returns to education for this group. Yahirun et al. (2017) and Ma (2019) also find that transfer payments from children to parents are serving as a pathway affecting parental health. These financial and material pathways are likely more relevant in poorer countries, where access to health care and, more importantly, healthy living conditions are contingent on financial resources. In Europe, intergenerational flows of material resources mostly go from the older generation to the younger generation (Albertini, Kohli, and Vogel 2007; Attias-Donfut, Ogg, and Wolff 2005), thus it is questionable how salient this financial support pathway in Great Britain would be. However, wealthier children could buy and help buy private health insurance for their parents.

Third, better education opens avenues into occupational fields that have direct health benefits to parents, namely, working in health care (Elo et al. 2018). This advantage can go indirectly via better information about health care as well as directly, namely, via preferential access and treatment. However, for Britain, with its universal and highly centralized health care system, it is unlikely that direct effects like preferential access are operating. However, the British health care system is very complex, and particularly low-educated parents could benefit from having children who know how to navigate it and to improve the doctor–patient relationship (Präg, Wittek, and Mills 2017). Doctors are highly educated and in authoritative positions; children with more education may be better able and more comfortable to communicate with doctors and to negotiate on behalf of their parents. Also, the fundamental causes perspective on health inequalities (Phelan, Link, and Tehranifar 2010) would argue that better-educated children are better able to support their parents in making use of resources that reduce preventable mortality.

Fourth, a stress pathway was recently proposed (Barr et al. 2018; Tosi and Albertini 2019). Barr et al. (2018) showed how problems in the transition to adulthood for Black adolescents (e.g., unemployment, romantic breakups, or arrests) heighten their mothers’ cumulative biological risk for chronic diseases. Thus, children leaving school early might be a stressful life event that has a toll on parents’ health. Moreover, successful offspring allows parents to invest fewer resources in financial and instrumental support to children. This implies that parental resources can be used to benefit the parents’ own health and material circumstances.

Fifth, shared genetic endowments might constitute a noncausal, confounding pathway linking adult children’s education to parental health. This genetic confounding occurs when the genotype—partially shared between parents and children—is associated with both children’s educational attainment and parental health (Krapohl et al. 2014). Marioni et al. (2016) showed that genetic variants associated with education are also linked to longevity. For example, parents of children with a strong genetic disposition for educational attainment lived half a year longer than parents of children with a weak disposition. Moreover, other sources of genetic confounding are also possible. Genetic endowments could drive selection into and exposure to individual and parental environment (Kong et al. 2018; Pingault et al. 2018). For instance, genes related to education might steer individuals into environments where health-promoting attitudes and behaviors are prevalent, and that, in turn, might enhance individuals’ and parental health. The presence of gene–environment correlation might suggest that social environment could be structured by genotype.

The effect of children’s education on their parents may differ by parental characteristics. Lower-SES parents may benefit more from having better-educated children because they have fewer resources. In their study on the Swedish population, Lundborg and Majlesi (2018) documented stronger effects of offspring’s education for lower-educated fathers. However, this is not generally the case. In their analysis of elderly Chinese, Yang et al. (2016) did not find any differences in having higher-educated children among the different educational groups of parents, while in an analysis of 11 European countries, Sabater and Graham (2016a) find mixed associational results depending on the gender of the parent and child.

## Data and Method

### Empirical Analysis 1: Cohort Study

#### Data: 1958 National Child and Development Study

The 1958 National Child Development Study (NCDS; Power and Elliott 2006) is a longitudinal cohort study following the lives of all 18,558 people born in England, Scotland, and Wales in the same week in 1958 from birth until now. Follow-up has been good over time; 84% of cohort members still participated at the age of 16, with a gradual decline in participation throughout adulthood—at age 55, 8,958 respondents participated in the study, yet in midadulthood, respondents were still broadly representative of the surviving cohort (Atherton et al. 2008). Next to collecting rich information about cohort members, the cohort study also asked respondents about the lives of their parents. We reconstructed parental longevity up to the most recent wave in 2013–2014, when cohort members were 55 years old.^2^

Covariates were measured as follows. Adult children’s education was based on a detailed educational qualification history, which was converted into school-leaving age entered as a continuous variable. This coding approach ensured similarity of our estimates with Empirical Analysis 2, which used school-leaving age to identify the causal effect of children’s education on parental health and mortality. For parents, school-leaving age of respondents’ fathers and mothers were reported by the parents in the 1974 wave, when respondents were age 16. We distinguished between four groups that reflect the most common educational qualifications in the parental generation. Age 13 to 15 years was equivalent to no qualification (reference category), age 16 to 17 years indicated lower secondary education, age 18 to 19 years denoted upper secondary or lower tertiary education, and age 20 years and more equaled a university degree. Parental social class stemmed from the same questionnaire and was based on parental reports of occupations, self-employment status, and supervisor status. We converted this to a seven-class version of the Erikson–Goldthorpe class schema^3^ with a dominance approach where we count only the highest class among the two parents (Connelly, Gayle, and Lambert 2016). We further accounted for the sex of the child (reference = male), whether the child was born out of wedlock (reference = in wedlock), and parental age at birth.

#### Method: Cox regression

To describe the association of adult children’s education and parental survival, we used the Cox regression model (Cox 1972), a semiparametric method that makes no assumptions about the functional form of the hazard. We estimated two models: The baseline model includes adult children’s education and the control variables sex, out-of-wedlock birth, and parental age at birth in the equation. The second model additionally controlled for parental social class and mothers’ and fathers’ school-leaving age. For both models, subjects entered the risk pool when parents were 45 years old.

Table 1 reports descriptive statistics concerning the children’s and parents’ characteristics. Only 45% of the mothers had died by 2013 to 2014, while 67% of the fathers had died. The median age at death was 69.7 years for mothers and 69.3 years for fathers. Maternal birth years ranged from 1910 to 1950, while paternal birth cohorts span from 1880 to 1942. Most of the cohort members were born to married parents; only 4% to unmarried ones.

**Table 1.** Descriptive Statistics Empirical Analysis 1.

	Proportion/Mean	SD
Mother’s average age at death	69.7	12.3
Mother deceased	.45	
Father’s average age at death	69.3	12
Father deceased	.67	
Female child	.51	
Child born out of wedlock	.04	
Mother’s average age at birth	27.46	5.73
Father’s average age at birth	30.57	6.45
Child’s age at leaving education	17.11	2.0
Number of child’s siblings	2.46	1.84
Father’s age at leaving education		
13–15 years	.58	
16–17 years	.30	
18–19 years	.07	
20+ years	.05	
Mothers’s age at leaving education		
13–15 years	.47	
16–17 years	.42	
18–19 years	.07	
20+ years	.04	
Parents’ social class		
I	.06	
II+IVa	.12	
III	.18	
IVb+c	.06	
V	.07	
VI	.21	
VII	.29	
*N*	11,710	

*Source:* National Child Development Study (Power and Elliott 2006), own calculations.

### Empirical Analysis 2: Linked Census Data

#### Data: the Office for National Statistics Longitudinal Study

We analyzed the Office for National Statistics Longitudinal Study (ONS-LS; Shelton et al. 2019), which combines linked census and life events data for a 1% sample of the population of England and Wales. The ONS-LS links records at each census since 1971 for people born on one of four selected (nondisclosed) dates in a year. Census information of the ONS-LS members is linked to life events data, including births, deaths, and cancer registrations. In 1971, women under the age of 60 who were married, widowed, or divorced were prompted to list the birth months and years of all live-born children who were born in wedlock. This birth date—expressed in three-month quarters around September 1—allowed us to assess whether children were affected by the 1972 educational reform in England and Wales.

Our analytical sample included all ONS-LS members who had children affected by the 1972 educational reform in England and Wales. It consisted of 56,199 mothers who had at least one child born between 1949 and 1965. In addition, we also analyzed the 49,612 male ONS-LS members whose wives reported their fertility history in the 1971 census. Table 2 describes the characteristics of the sample stratified by parental sex.

**Table 2.** Descriptive Statistics Empirical Analysis 2 for Office for National Statistics Longitudinal Study Sample Members (Parents).

	Men	Women
Education in 1971		
% No qualification (lower educated)	85.4	90.9
% A level (higher educated)	8.9	7.9
% Higher than A level (higher educated)	5.6	1.1
% Having children affected by reform	51.9	51.1
Regional deprivation		
% First quintile (least deprived)	19.6	19.1
% Second quintile	21.6	21.3
% Third quintile	21.0	21.7
% Fourth quintile	20.1	20.7
% Fifth quintile (most deprived)	17.7	17.2
Social class		
% I/II social class	25.3	23.3
% III nonmanual social class	9.7	10.6
% III manual social class	40.2	35.6
% IV/V social class	22.1	22.1
% Unclassified social class	2.7	8.4
Mean age at first birth	29.9	27.2
*n*	49,612	56,199

*Source:* ONS-LS (Shelton et al., 2019), own calculations.

We analyzed a range of both objective and subjective health outcomes. We examined mortality and premature mortality (before age 65). Respondents were followed until December 31, 2015, to assess their mortality status. Furthermore, we distinguished between six causes of death based on the ICD classification, namely, lung cancer, accidents and self-harm, liver disease, ischaemic heart disease, mental and behavioral causes, and preventable causes in general (we defined causes as preventable following the definition in Office for National Statistics 2011). These causes of death were selected because of their strong association with some health behaviors, such as smoking and excessive alcohol consumption. The aim was to test offspring’s influence through parental healthy lifestyle behaviors. Furthermore, we examined different self-reported health outcomes, namely, self-reported long-standing illnesses, measured in 1991, 2001, and 2011, and self-reported poor general health, measured in 2001 and 2011.

We included the following control variables in our models: parent’s year of birth, dummy variables for parental occupational class, education (measured as in whether the parents attained A-level education, a UK qualification corresponding to 13 years of schooling), and number of children. Occupational class is indicated by the registrar-general’s class scheme (Connelly et al. 2016), comprising Classes I/II (“Professional occupations” and “Managerial and technical occupations”), III nonmanual (“Skilled nonmanual occupations”), III manual (“Skilled manual occupation”), and IV and V (”Partly-skilled occupations” and “Unskilled occupations”). Class was derived from respondent’s occupation in 1971. Women married at the time of the census have been assigned the occupational class of their husband if a husband was present; otherwise, their own occupational class.

The parents in our sample can of course have multiple children who may or may not have been affected by the reform. Therefore, we created a data set where each case referred to one child (rather than one parent), and we conducted a child-level analysis because each parent can have several children, of which more than one can be affected by the reform. To account for this, we weighted our estimates by the inverse of the number of children a parent has, following the approach in Lundborg and Majlesi (2018).^4^

#### Method: regression discontinuity approach

Using a regression discontinuity design, we exploited the exogenous variation in age at school completion due to compulsory schooling law changes in 1972 in England and Wales. The intuition behind a regression discontinuity design is to compare cases just below and above a fixed cutoff value that determines whether cases receive the treatment. The assumption is that these cases just below and just above the threshold differ only in whether they receive the treatment but are (on average) the same on both observed and unobserved variables. This section first describes the educational reform at the center of the analyses and then describes our estimation approach.

#### The 1972 educational reform in England and Wales

Compulsory schooling laws in Britain define the maximum age at which children should start education and the minimum age at which they are allowed to leave education. Consequently, these laws determine how many years children spend in compulsory schooling. During the twentieth century, two legal changes increased the minimum ages at which children were allowed to leave school in England and Wales. First, the 1944 Educational Act, the “Butler Act,” established an increase in school-leaving age from 14 to 15. This change was implemented from April 1, 1947, onward. Second, the same Education Act authorized a further increase in school-leaving age from 15 to 16, which was implemented starting from September 1, 1972. This is the reform that we used for our study because it falls in the observation period of the 1971 census and allows exploring causal effects of children’s education on parental mortality in different time periods.

These schooling reforms have been exploited in previous research, but none has examined the intergenerational effects in which we are interested. Clark and Royer (2013), Davies et al. (2018), and Grenet (2013) found that the 1972 reform increased earnings for both men and women, suggesting plausible spillover effects on parents through financial resources. Davies et al. (2018) further showed that the reform significantly reduced blood pressure, diabetes, heart attack risk, body mass index, and sedentary and unhealthy behaviors. However, not all studies show positive effects of the 1972 reform on health and health behaviors and knowledge (Clark and Royer 2013; Janke et al. 2020; Johnston et al. 2015; Silles 2009). For example, Jürges, Kruk, and Reinhold (2013) did not find any effect on self-rated health and some biomarkers, such as level of fibrinogen and C-reactive protein. Similarly, Powdthavee (2010) showed that the 1972 reform did not decrease significantly the likelihood of hypertension. Despite the unclear effect of the 1972 educational reform on the health of the affected cohorts, it remains important to study the spillover effects of children’s education on parental health and mortality. The mechanisms linking children’s education to parental health that we have hypothesized are not necessarily dependent on children’s health but could also operate via behaviors and earnings, which have been found to be affected by the educational reform (Clark and Royer 2013; Davies et al. 2018; Grenet 2013).

**Figure 1.**
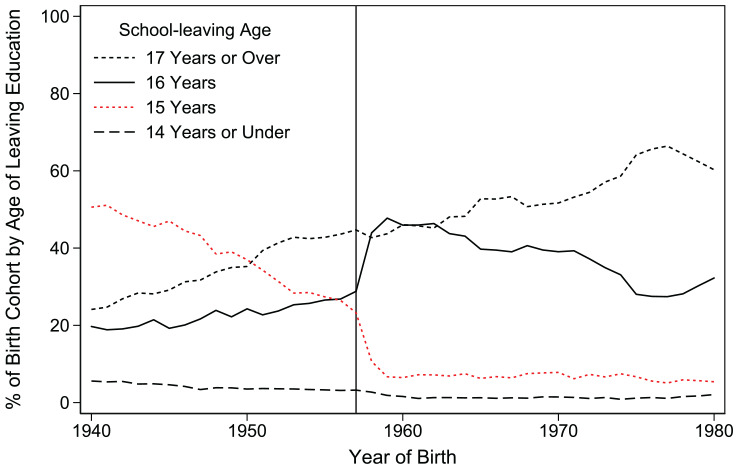
The 1972 Educational Reform Greatly Reduced the Share of Individuals Leaving School at Age 15 for Those Born after September 1st, 1957. *Source:* Health Surveys for England, 1991–2013 (Mindell et al. 2012), own calculations. *Note:* Years of full-time education by year of birth.

Figure 1 shows the increase in school-leaving age across birth cohorts in England. The 1972 reform had a clear effect in producing a discontinuous increase in years of education across birth cohorts. Furthermore, we can see that compliance with the reform is high. The reform reduced the percentage of individuals with only nine years of schooling by almost 17 percentage points. Next to that, the reform also did not affect the percentage of individuals with more than 16 years of education. We relied on the Health Surveys for England (1991–2013) for demonstrating this discontinuity because we knew only the quarter of birth, not the actual educational attainment of the children of ONS-LS sample members.

#### Identification strategy

Identification was achieved through a regression discontinuity (RD) approach (Imbens and Lemieux 2008; Lee and Lemieux 2010). RD designs have four main features: the running variable, the treatment, the cutoff, and the bandwidth. The running variable was the children’s date of birth, measured in quarter of the year (due to confidentiality restrictions imposed by the data provider), that lead to the assignment of the treatment. Second, the treatment was the increase in years of compulsory education for individuals born after September 1, 1957. The cutoff represented the quarter of birth after which the treatment was assigned. Therefore, the probability of treatment assignment based on the running variable had a discontinuous change after the reform implementation. Finally, the accuracy of estimation depended on the bandwidth (i.e., the number of observations right before and after the reform threshold). It was crucial that the bandwidth estimation procedure was conducted in a data-driven way such that the trade-off between bias (i.e., when the bandwidth is large, the larger is the bias) and variance (i.e., when the bandwidth is small, the larger is the variance) is minimized.

Our design was a “fuzzy” (as opposed to “sharp”) RD approach because the reform increases the probability to stay in education until age 16 but does not solely determine whether a student stays in school. All the analyses were carried out with the Stata package *rdrobust* (Calonico et al. 2017). We used a data-driven bandwidth estimation procedure (using the same mean square error optimal bandwidth on both sides) and checked the sensitivity of the results to different bandwidths and bandwidths’ selection algorithm. Following Gelman and Imbens (2019), we chose a linear fit for the forcing variable and calculate robust standard errors. We added a number of control variables (listed previously) to our models. We included control variables in our models because they increased the precision of the estimates by removing small residual unbalances between groups.

Using a RD design to study the effects of a policy reform has an advantage over studies relying on instrumental variable (IV) approaches to estimate causal effects. IV methods in quasi-experimental settings can be sensitive to cohort trends in mortality. In particular, small effects of offspring’s education on parental mortality due to an educational reform are more difficult to distinguish from secular changes in mortality when an IV approach is used (Galama et al. 2018). Our RD approach allowed us to address such a bias.

We compared cases with children born right before and after the cutoff date. For illustration, Table 3 shows outcome variables and parental covariates for children born one year before and one year after the reform. The groups were balanced for the covariates, supporting the assumption of quasi-random assignment to the reform, but we observed some differences for the outcomes. The actual sample size in the models of the different outcomes depended on bandwidth used and the number of nonmissing observations for each outcome. We conducted an ex-post power analysis using the *rdpower* command in Stata (Cattaneo, Titiunik, and Vazquez-Bare 2019) to check whether a lack of statistical power can account for our results. The power analysis concluded that we have more than enough power (>.8, which is the customary threshold for power) to detect effect sizes half of a standard deviation of the outcome for the untreated and even smaller effect sizes for many of the outcomes. Detailed information about the sample size for each estimation and the results from the power analysis are available in the Appendix, Sections C.2 and D.2, available in the online version of the article.

**Table 3.** Descriptive Statistics for Outcome Variables and Covariates for Parents with Any Children Born between One Year before and One Year after the Reform.

	Without Children Affected by Reform	With Children Affected By Reform
Outcome variables		
% Deceased until December 31, 2015	62.1	55.9
% Deceased before age 65 until December 31, 2015	14.2	14.7
% Died of lung cancer	5.4	4.6
% Died of accidents and self-harm	1.0	1.0
% Died of liver disease	.8	.7
% Died of ischaemic heart disease	11.3	8.9
% Died of mental and behavioral causes	1.5	1.6
% Died of preventable causes	8.0	7.9
% Long-standing illness in 1991 census	24.3	21.7
% Long-standing illness in 2001 census	43.3	41.4
% Long-standing illness in 2011 census	63.6	60.5
% Poor self-rated health in 2001 census	20.9	18.9
% Poor self-rated health in 2011 census	18.1	16.8
Parental covariates		
% Women	52.6	52.5
Average number of children	2.7	2.6
Average parental year of birth	1928	1930
% Higher educated	12.3	12.7
Social class		
% I/II	25.3	25.6
% III nonmanual	9.9	9.9
% III manual	38.0	37.6
% IV/V	20.9	21.7
% Unclassified	5.7	5.5
*n*	6,089	6,057

*Source:* Office for National Statistics Longitudinal Study (Shelton et al. 2019), own calculations.

The estimated treatment effects represent intention-to-treat (ITT) effects, which are the causal effects of children’s reform eligibility defined by the threshold rule (i.e., born after September 1, 1957) on parental health and mortality outcomes. ITT effects use the quasi-random assignment to the policy reform (because individuals cannot manipulate their date of birth) and thus are the average impact of the reform both for those who actually stay one additional year in compulsory education and for those who do not. How does an ITT effecxt differ from the average treatment effect (i.e., the effect of one additional year of education who actually stayed in school one year longer)? The ITT effect does not take into account noncompliance (i.e., individuals who drop out of school before reaching the mandatory school age despite the reform), making it potentially smaller than the average treatment effect. Yet previous studies have found very few people leaving school before compulsory age after the reform we build our analysis on (Clark and Royer 2013; Silles 2009). Therefore, we expected that our ITT effect is not substantively different from the average treatment effect. Data restrictions made it impossible to estimate the causal effect on those who comply to the policy reform (i.e., only those who stay one year more in compulsory schooling) because the linked census data contained only the date of birth but not the age at school leaving or educational attainment as children’s information.

## Results

### Empirical Analysis 1: Cohort Study

**Figure 2.**
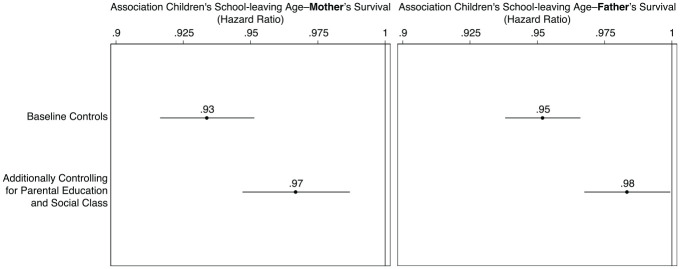
Better-Educated Children Have Parents Who Live Longer, Even When Comparing Parents from the Same Social Class and with the Same Educational Attainment. *Source*: National Child Development Study (Power and Elliott 2005), own calculations. *Note:* Hazard ratios are based on the models reported in Table A2 in the online version of the article. Spikes denote 95% confidence intervals.

Figure 2 presents the results from the Cox proportional hazard models and confirms the association between children’s education and parental mortality. Children who have left school later have longer-living parents. In the baseline model (top row), one additional year of children’s education is associated with a hazard ratio for maternal death of .93 (95% confidence interval (CI) = .92 to .95) and for paternal death of .95 (95% CI = .94 to .97), corresponding to a 6% and 5% decrease in the expected hazard.

Once we add parental social class and parental educational attainment to the equation, the association is attenuated, yet the overall pattern holds and remains statistically significant at conventional levels. Having a child who left education at older age is associated with a 3% decrease in the expected hazards of death for mothers and 2% for fathers. This is a substantially important association because predictions of median years of life lost based on the second models suggest that parents of less-educated children (i.e., leaving school at age 16) have a two-year difference compared to parents of more educated ones (i.e., leaving school at age 21). This difference is similar to predictions in previous studies reporting about two years difference in life expectancy (Friedman and Mare 2014).

Several robustness checks were carried out. First, as shown in Table A4 (available in the online version of the article), we assessed the role of missing values by comparing the results to those obtained from using multiple imputation with chained equations (White, Royston, and Wood 2011). Second, as shown in Table A4 (available in the online version of the article), we restricted the sample to birth cohort members who lived in England and Wales at age 16 (excluding those living in Scotland) to make it more comparable to the data used in Empirical Analysis 2. Third, as shown in Table A5 (available in the online version of the article), we included an interaction of children’s education with time to assess sensitivity to violation of the proportional hazard assumption. Fourth, as shown in Table A5 (available in the online version of the article), we used an accelerated failure model with survival time following a Weibull distribution. In all cases, the magnitude of the coefficients changed slightly, but the direction of the associations stayed the same.

### Empirical Analysis 2: Linked Census Data

**Figure 3.**
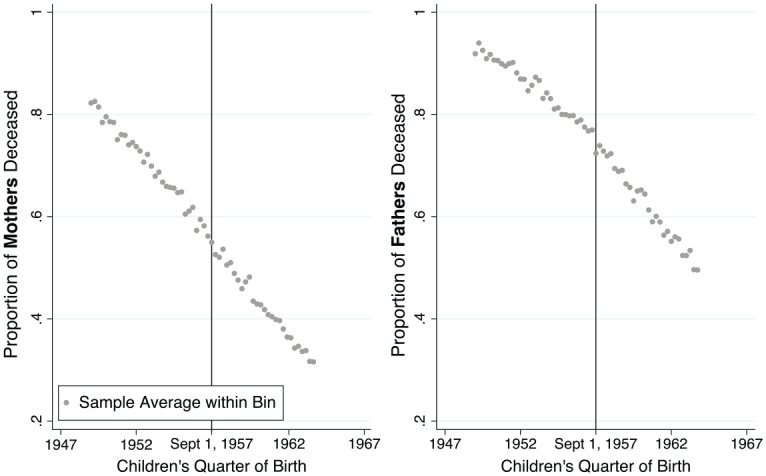
The Proportion of Deceased Parents Decreased Approximately Linearly over a Period Spanning Two Decades. *Source*: Office for National Statistics Longitudinal Study (Shelton et al. 2019), own calculation.

**Figure 4.**
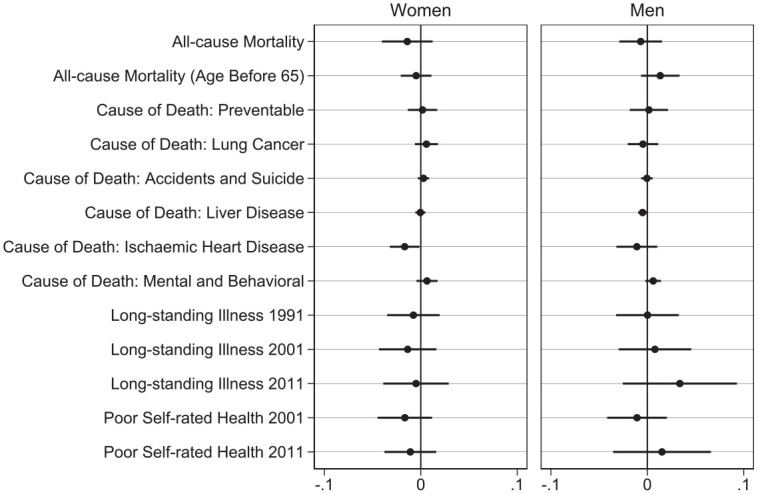
Regression Discontinuity Estimates of Educational Reform Stratified by Parental Sex. *Source*: Office for National Statistics Longitudinal Study (Shelton et al. 2019), own calculations. *Note*: Error bars denote 95% confidence interval based on robust standard errors. Controls comprise parental education, number of children, occupational class, and year of birth. Estimates shown in Table A3, available in the online version of the article.

The previous section established the presence of associational findings, while this second part of the analysis aimed at determining whether there is a causal effect. Figure 3 shows the proportion of parents who have died before 2016 by children’s quarter of birth. Qualitatively, the mother’s curve does not reveal any appreciable discontinuity, while the father’s mortality presents candidate features around the time of the educational reform. The statistical significance of the jump is analyzed and shown in Figure 4.

Figure 4 presents the results from regression discontinuity analyses for all 13 outcomes, stratified by parental sex. The coefficients do not always point in the expected direction and usually do not reach conventional levels of statistical significance. Mothers with children affected by the reform experience a small reduction in mortality. Having a child affected by the reform reduces the probability of dying by 1.4%, but this estimate does not reach conventional levels of statistical significance (95% CI = −4.0 % to 1.2 %). For fathers, the coefficient from the model for overall mortality points in the same direction ( −0.7 %; 95% CI = −2.9 % to 1.5 %), but similarly, it is not significant at conventional levels. Two estimates do reach conventional levels of statistical significance: for women, the risk of dying of ischaemic heart disease and for men, the risk of dying of liver disease. However, after correction for multiple comparisons with the Benjamini–Hochberg procedure for the six causes of death considered here, neither of these results is significant.

We further stratify the models by the socioeconomic position of the parents. Results show that there is no systematic difference by parental socioeconomic position in the effect of children’s education on parents’ outcomes. Results can be found in Figure A3 in the online version of the article. We conducted several robustness checks. First, we check that the results in Figure 4 are robust to multiple comparison correction using the Benjamini–Hochberg procedure for multiple tests in the causes of death considering them as independent outcomes. Results show that none of the coefficients remain significant after this adjustment. Second, as shown in Figure A4, available in the online version of the article, we stratified the models by parental education and by area deprivation as two additional measures of parental class. While a number of estimates are statistically significant (at the 5% level), they are specific to single SES indicators, which does not support a notion that lower-SES parents benefit more from better-educated children. Third, as shown in Table A7, available in the online version of the article, we stratified models by the number of children that parents had. Effects might be especially pronounced among parents whose only child was affected by the reform rather than among parents with more children, some of whom will not be affected by the reform. This robustness check did not reveal any significant effects, which is in line with our overall findings. Finally, as shown in Table A10, available in the online version of the article, we investigate the effects of choosing different bandwidths around the threshold date. The results remain the same when different bandwidths are selected, demonstrating low sensitivity to the bandwidth chosen.

## Conclusion

This study explored the intergenerational consequences of education for population health in Great Britain. First, we establish that there is an association between adult offspring’s education and parental mortality in Great Britain, which exists even when comparing only parents with the same occupational class background and the same educational attainment. Specifically, having a better-educated child increases median parental longevity by two years. Second, using an educational reform as a natural experiment, we analyze linked census data from England and Wales to show that the increase of children’s school-leaving age from 15 to 16 had no significant causal effect on parental health, overall mortality, or specific causes of death. An investigation into heterogeneous treatment effects by parental socioeconomic status shows that health benefits do not accrue particularly for parents from disadvantaged backgrounds regardless of indicator of disadvantage we use.

In the first analysis, we find a significant association between adult children’s education and parental longevity. From our second analysis, we conclude that this association does not result from a direct causal effect of education on parental health using a local experiment. We propose the absence of a causal effect in our study might be explained by three aspects in which the British context is unique: universal health care coverage, the relevance of education for determining socioeconomic position, and the location of the change in education brought about by the 1972 schooling reform. First, we have to take into account the potentially equalizing effect of the National Health Service (NHS) in Great Britain. The NHS provides universal and comprehensive access to health care free at the point of use and covers all health care needs over the entire life course. This single-payer health care system potentially removes financial barriers to accessing health care, making parents rely less on their children’s economic resources for support.

Second, a possible explanation could be that in Great Britain, occupational class—rather than education—is particularly salient for life chances, especially compared to countries such as the United States, where education is a more important driver of life chances. Parental occupational class of origin is a major predictor of children’s pay, even in professional and managerial occupations in the United Kingdom (Laurison and Friedman 2016). Previous studies have shown the effect of the reform has increased the probability of working in nonmanual occupations, in particular, in the sectors of public administration, health, and education (Lepinteur and Nieto 2019).

Third, the educational reform increased school-leaving age from 15 to 16 years, which, although an important increase in overall educational level of the population and with documented effects on income, might not have been salient enough for an overall health effect. However, it is the least advantaged young people who could have benefited the most from the additional year in compulsory schooling. Therefore, we expect that the policy reform would have affected the intergenerational life chances of the most disadvantaged families. Moreover, there might be causal effects of offspring’s education at other points of the educational distribution, for example, receiving college education compared to having only secondary education. Given our focus on a quasi-natural experiment that raised the mandatory school leaving age from 15 to 16, we could not establish effects at other points in the educational distribution. Madia, Präg, and Monden (2022) expands our analyses to include a reform that increased the school-leaving age from 14 to 15 years of age. Reforms in other settings that affected tertiary education would be a tool to identify such effects.

We find no evidence for low-SES parents benefiting more strongly from their children’s education. This is a particularly striking finding because we would expect to find the strongest effect for low-SES parents. The only other causal study in a resource-rich setting, examining an educational reform in Sweden (Lundborg and Majlesi 2018), found effects for the health of lower-educated parents only, not for higher-educated parents. However, these effects materialized only for specific gender combinations, namely, for fathers of daughters, which we cannot investigate with the data at hand.

Our study is not free from limitations. First, while it would be interesting to study additive effects of children education on parental health, due to the very reduced number of multiple births in the data set, this analysis could not be carried out. Second, the absence of a causal effect has to be understood locally, and therefore, we cannot exclude the presence of an effect at other points in the educational distribution. Third, the data in hand did not allow us to make direct tests of potential causal mechanisms linking adult children’s education and parental longevity, such as learning of improved health behaviors, increased material resources, and reduced stress. Finally, parents in the first NCDS analysis are known to have some contact with their children (who respond to the survey at age 45); the second study uses ONS-LS census-linked data, which does not require any direct contact with the children after the birth of child.

The fundamental causes perspective on health inequalities (Clouston and Link 2021) posits that socioeconomic inequalities arise when it is possible for more socioeconomically advantaged individuals to obtain health benefits by leveraging resources available to them. In the context of linked lives, the theory expects that the more preventable a disease is, the more responsive it would be to an increase in offspring’s education. However, we compared different causes of death that should be particularly prone to improve with children’s education without finding any effect. This absence of consistent evidence for other causes of death or health indicators limits our ability to draw definitive conclusions on this mechanism.

Therefore, improving the education of younger generations might be a useful policy instrument with intergenerational benefits in the context of aging populations. Such an educational expansion might not just improve the human capital of younger generations but also have positive spillover effects that improve the health of older generations. Our study is the first to comprehensively test the causal effect of adult children’s education on a comprehensive set of health and mortality outcomes in a high-income country with an aging population. Our results suggest the presence of a significant association between children’s education and parental health. However, neither parental health nor longevity is affected by children’s exogenous increase in education, casting doubts on the intergenerational benefits of human capital investments.

## Supplemental Material

sj-docx-1-hsb-10.1177_00221465221143089 – Supplemental material for Does Children’s Education Improve Parental Health and Longevity? Causal Evidence from Great Britain Click here for additional data file. Supplemental material, sj-docx-1-hsb-10.1177_00221465221143089 for Does Children’s Education Improve Parental Health and Longevity? Causal Evidence from Great Britain by Cecilia Potente, Patrick Präg and Christiaan W. S. Monden in Journal of Health and Social Behavior

## Notes

1. The census and cohort data sets use parent-centric and child-centric focal points, respectively. While most studies connecting adult children’s education and parental health have been based on parent-centric data, both focal points are equivalent for the analyses conducted here.

2. The 2004 wave of the NCDS did not collect information about the exact timing of parental death. For parents who deceased between the 2000 wave and the 2004 wave (1,209 fathers and 879 mothers), we thus do not know the exact timing of death and hence imputed a random year of death for the cases in question. Robustness checks showed that this did not affect our results. Cohort members’ fathers and mothers were on average born in 1927 (SD = 6.4 years) and 1931 (*SD* = 5.7 years), respectively. We retained 11,710 cases for which parental survival was possible to record from the 1999/2000 wave—when parental survival was first probed—and who remained in the sample at least until the 2013 to 2014 wave or until their parents decease.

3. (I) Professionals, administrators and managers, higher-grade (reference category); (II+IVa) professionals, administrators and managers, lower-grade, small employers, higher-grade technicians; (III) routine nonmanual employees; (IVb+c) self-employed workers (including farmers); (V) lower-grade technicians, supervisors of manual workers; (VI) skilled manual workers; (VII) nonskilled manual workers.

4. Parents with multiple children were accounted for multiple times in the analysis. To prevent the resulting overrepresentation of larger families, we thus weighted the estimates by the inverse of the number of children. This weighting by the number of children ensured that every parent is accounted for only once.
