# Supplementary material for: Does Children’s Education Improve Parental Health and Longevity? Causal Evidence from Great Britain
Source: J Health Soc Behav. 2023 Jan 27;64(1):21–38. doi: 10.1177/00221465221143089 (PMC10009472; doi:10.1177/00221465221143089)
Supplement: sj-docx-1-hsb-10.1177_00221465221143089 – Supplemental material for Does Children’s Education Improve Parental Health and Longevity? Causal Evidence from Great Britain [file sj-docx-1-hsb-10.1177_00221465221143089.docx]

**Journal** of **Health**

and **Social Behavior**

OFFICIAL JOURNAL OF THE AMERICAN SOCIOLOGICAL ASSOCIATION

**ONLINE SUPPLEMENT**

**to article in**

Journal of Health and Social Behavior

**Does Children’s Education Improve Parental Health and Longevity? Causal Evidence from Great Britain**

**Cecilia Potente**

*University of Zurich*

**Patrick Präg**

*CREST, ENSAE, Institut Polytechnique de Paris*

**Christiaan Monden**

*University of Oxford*

# A Replication materials and data references

Analyses shown in this manuscript are fully replicable. Program code for the replication will be available on-line.

Data used in the study are publicly available. The birth cohort data analyzed in this study (Power and Elliott, 2006) as well as the data used for Figure 3 (Mindell *et al.*, 2012) are available from the UK Data Service to registered researchers. The linked census data analyzed in this study (Shelton *et al.*, 2019) are available from the Office for National Statistics (ONS) to accredited researchers after the opening of an approved research project. Further information on how to obtain accredited researcher status is available from the Office for National Statistics.

**B Descriptive analysis of all 27 existing studies on adult children’s education and parental health**

Figure 1: Descriptive analysis of all 27 existing studies on adult children’s education and parental health. *Panel A*: Research on offspring education and parental health is flourishing in the last five years. Panel B: Studies are focused on a small number of countries, mostly the US. *Panel C*: Associational study designs make up the bulk of studies. *Panel D*: Most studies focus on mortality or wellbeing/mental health outcomes.

Note: Studies included in the literature review listed at the end of the document. Some studies report more than one outcome variable.

# C Question wording for longstanding illness and poor self-rated health in ONS-LS (Shelton *et al.*, 2019)

The census question wording for longstanding illness and poor self-rated health has varied over time, in Table A1 we show the exact wording of the question and response options. Parentheses behind response options show how we coded the responses for analysis.


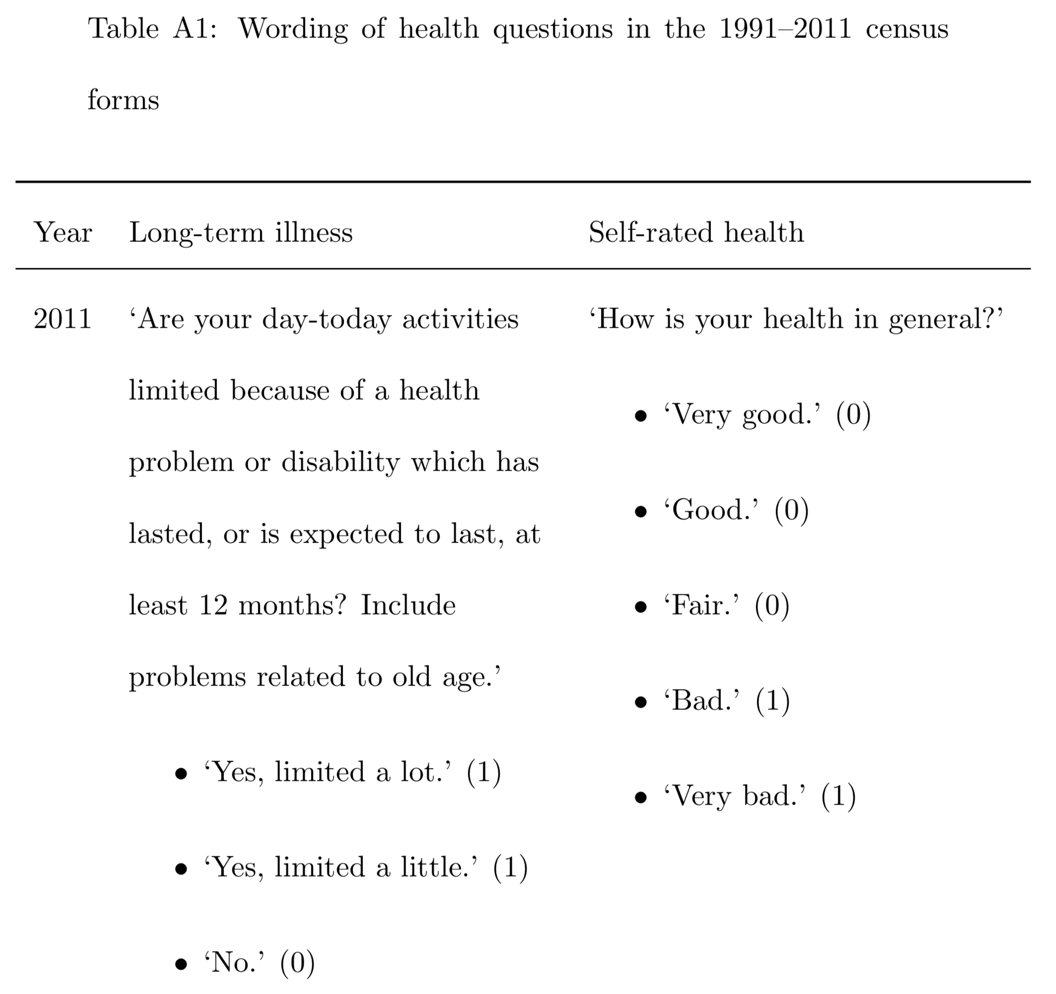


#
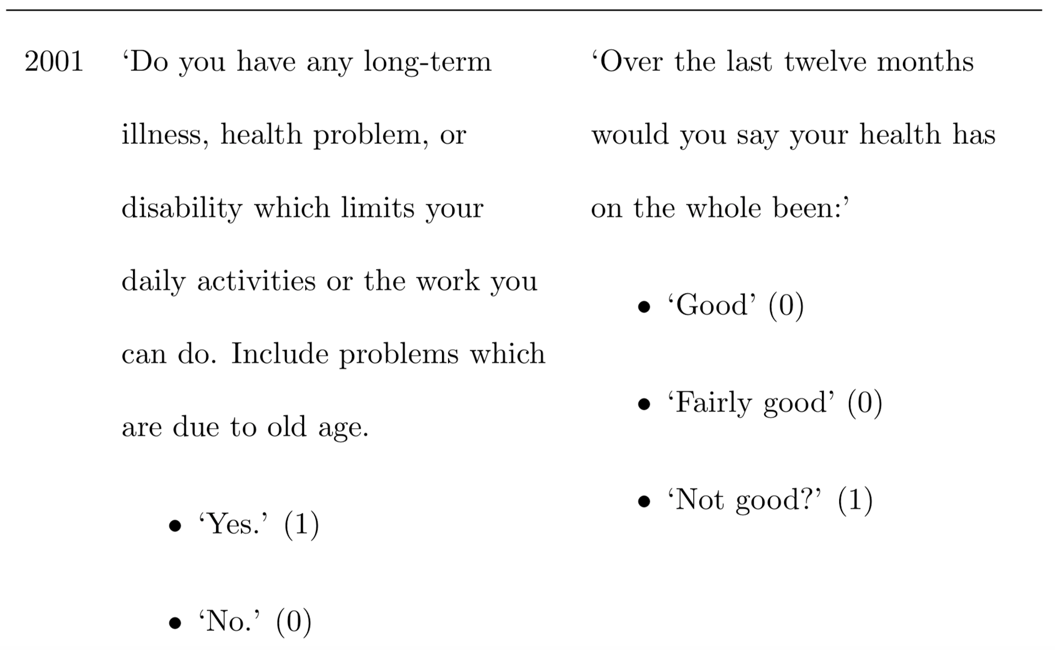


#
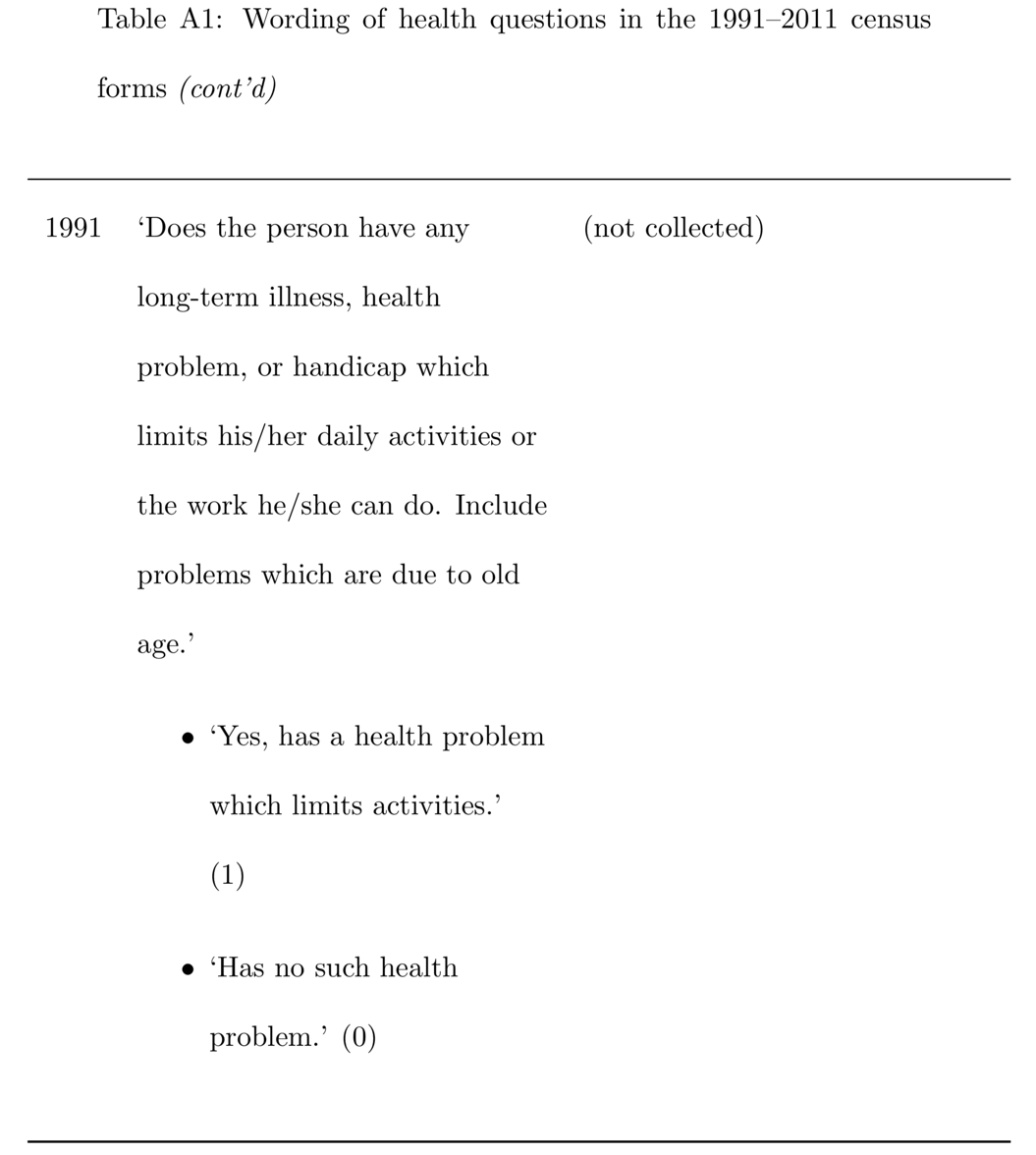


# D Tables underlying Figures in the main text

**D.1 Empirical analysis 1: birth cohort**

Table A2 shows the full models underlying Figure 1 in the main text.


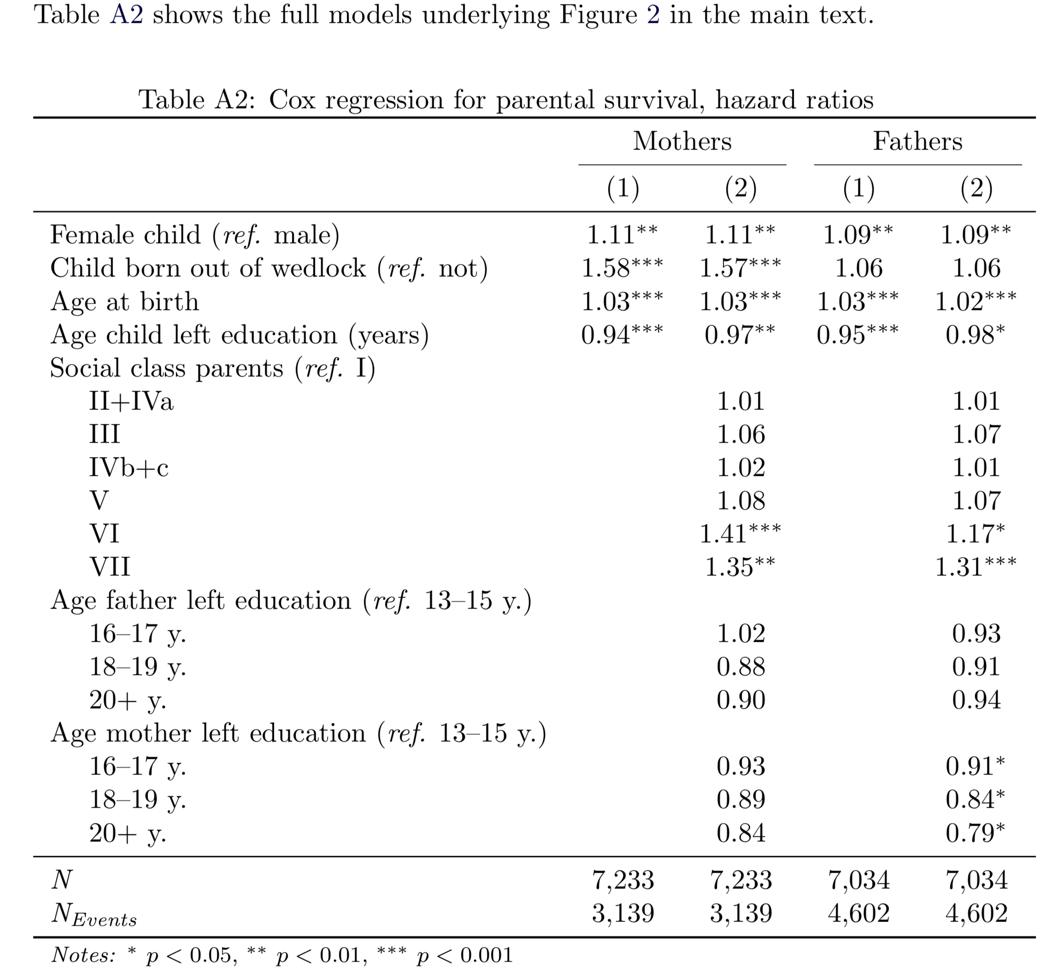


Source: NCDS (Power and Elliot, 2006)

**D.2 Empirical analysis 2: linked census data**

Tables A3 shows estimates underlying Figure 4 in the main text. We also show the results for an additional stratification by sex and parental occupation in Table A4.


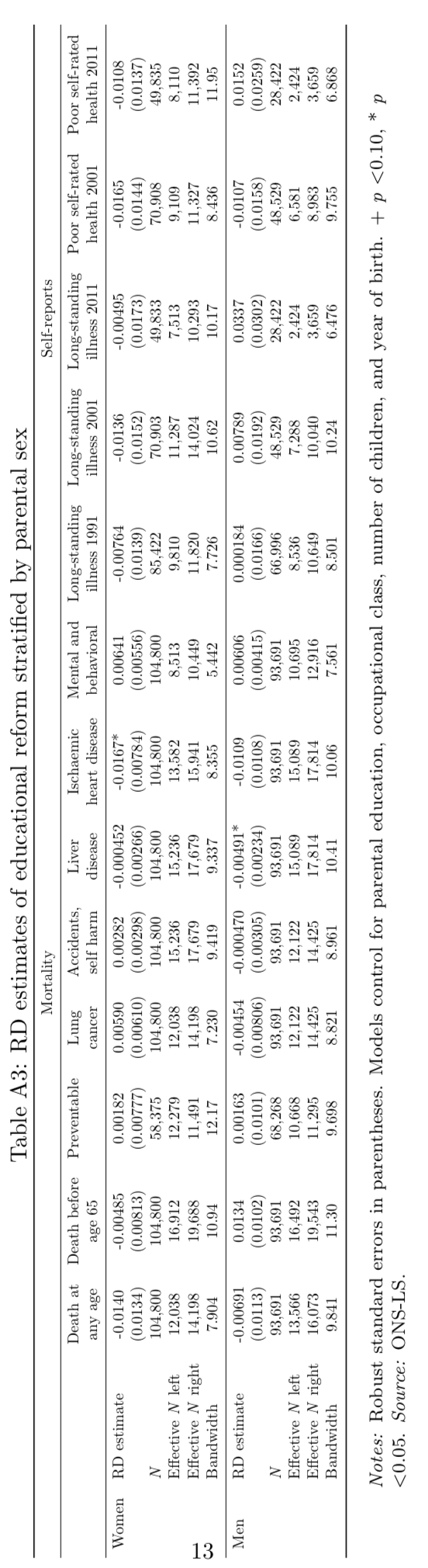


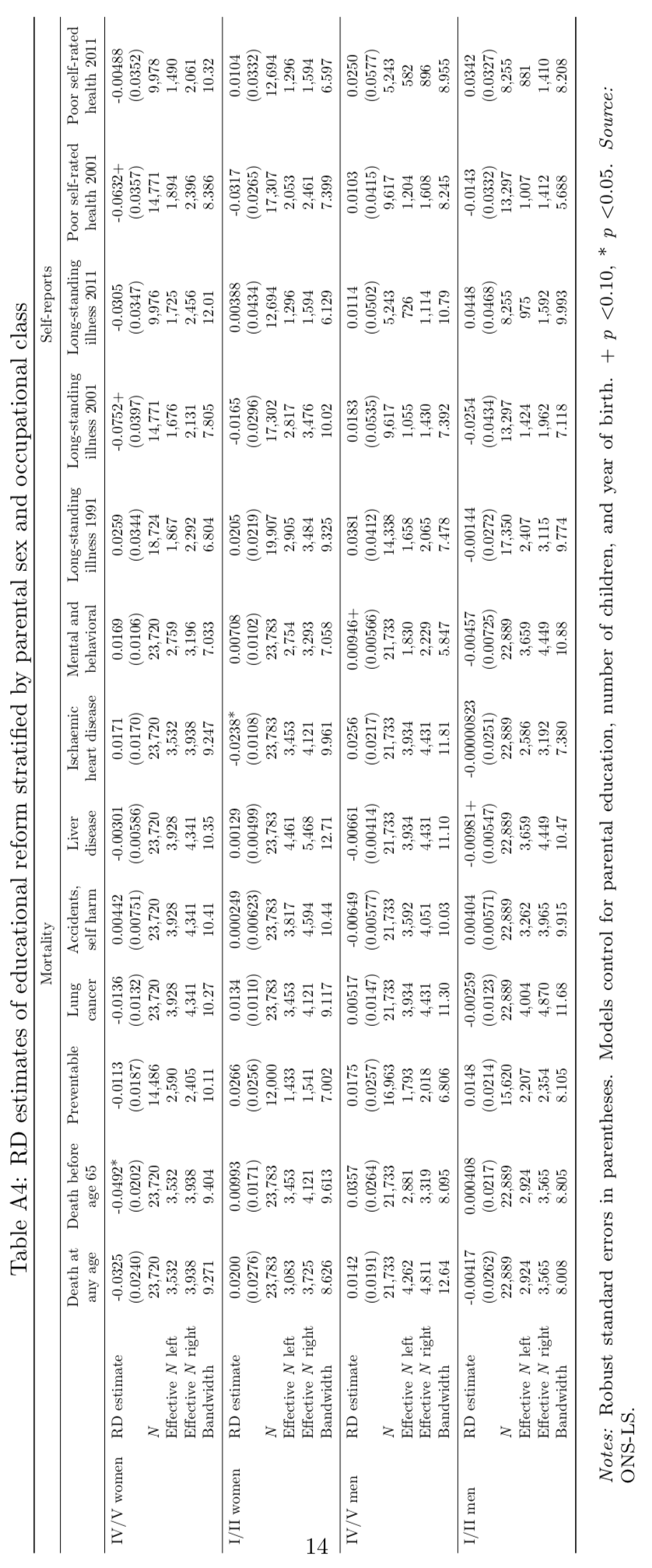


# E Robustness checks

**E.1 Empirical analysis 1: birth cohort**

In Table A5 and A6, we show four key robustness checks of our results.

First, we assessed the role of missing values by comparing the results to those obtained from using multiple imputation with chained equations (MICE, White *et al.*, 2011). We imputed the following variables: mother’s/father’s age at birth with linear regression, child sex and child born out of wedlock with a logit link, education of the father, mother, and child with an ordered logit link, and parental social class with a multinomial logit link. We use as auxiliary variables the indicator dummy for the death of the father and the mother plus the Nelson–Aalen estimate of the cumulative hazards for fathers and mothers, as suggested by White and Royston (2009). For determining *m*, the number of imputations, we made use of Von Hippel’s (2020) algorithm, which suggested between 3 and 37 imputations for our models. We used 37 imputations for every model. We ran models on the imputed data and combined the resulting estimators using the rules stated by Rubin (1987, ch. 3).

Second, we restricted the sample restricted to birth cohort members who were living in England and Wales at age 16 (excluding those living in Scotland) in order to make it comparable to the second part of the analysis which focuses on a schooling reform that only took place in England and Wales. In all cases, the magnitude of the coefficients changes slightly but the direction of the associations stays the same.

Third, we assessed the proportional hazard assumption using Schoenfeld residuals, which suggested that the assumption might be violated for the adult children’s education variable. Substantively, this means that the association of children’s education and parental longevity varies over time and that the associations shown in Figure 2 show a rough average of the strength of the association over time. Models including the interaction of children’s education with time however lead to the same substantive conclusion of an association between children’s education and parental longevity, which is attenuated when accounting for parental class and education.

Fourth, we further test the robustness of our results using a parametric acceleration failure time survival model following a Weibull distribution. This model offers an alternative to the Cox model by assuming that the effect of the covariates is proportional to the survival time (and not to the hazard as in the Cox model). Using this specification the time ratios associated with an additional year of children’s education shows an increase in survival by 1.4 % for mothers and by 0.8 % for fathers in the full covariates model.

Fifth, we test the robustness of the results to families with more than one offspring by including an indicator of whether the respondent is single child or not at age 16. Unfortunately, the data do not contain the educational level for the other siblings and therefore we could not perform additional robustness checks on different operationalizations for the educational level of the siblings. The results presented in Figure 2 shows similar conclusion as in the main analysis.

Finally, we include a robustness test of the results using categorical education variable to account for a possible non-linear correlation. The results, shown in Figure 3, provide similar conclusion to the linear analysis.

Figure 2: Cox proportional hazard models for the association between children’s education and parental mortality including binary variable indicating whether the child is single child or not.

Source: NCDS (Power and Elliot, 2006)


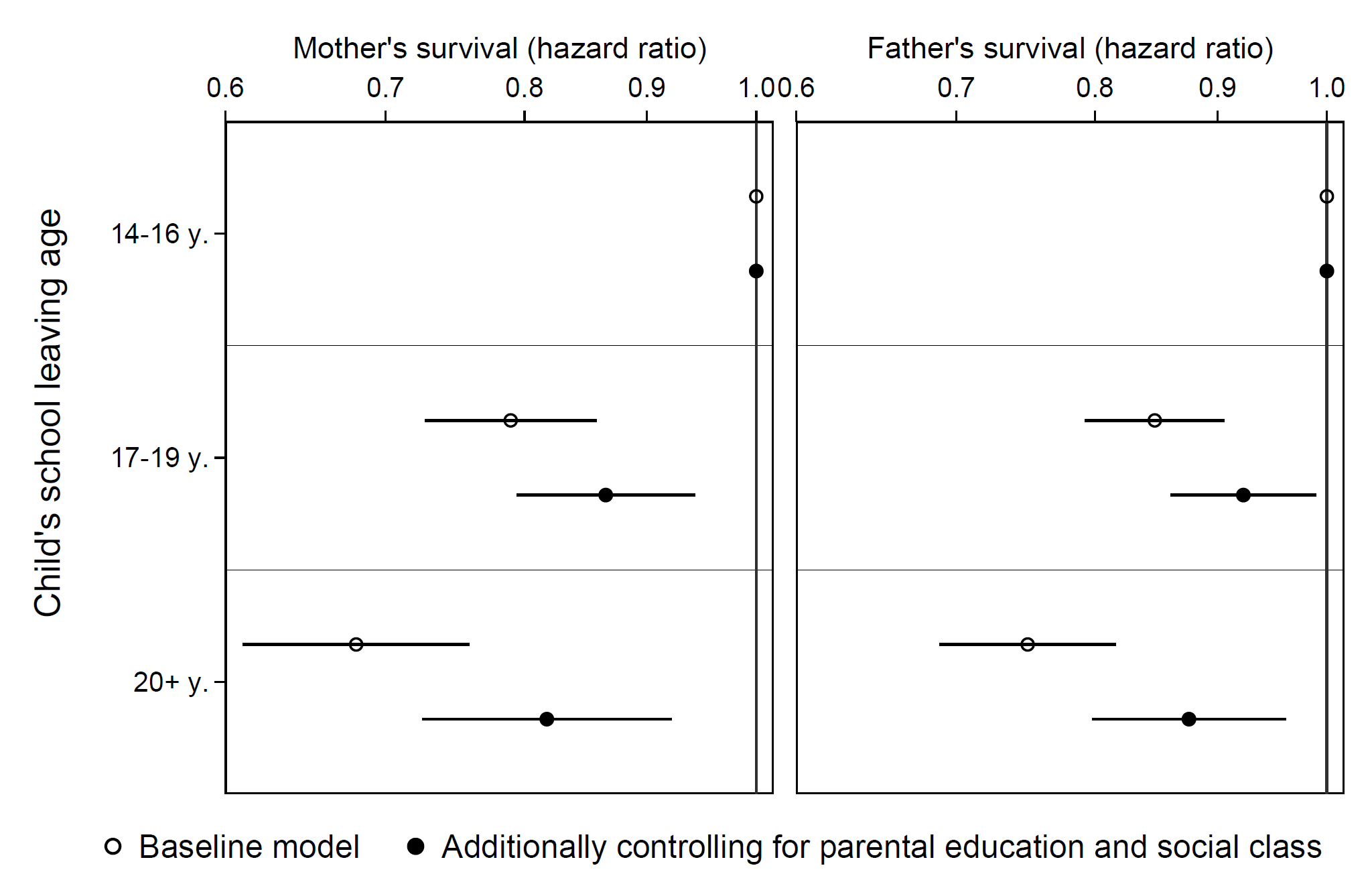


Figure 3: Cox proportional hazard models for the association between children’s education and parental mortality using categorical specification of children’s education.

Source: NCDS (Power and Elliot, 2006)

**E.2 Empirical analysis 2: linked census data**

**Education and area deprivation as stratifying variables** Figure A1 as well as Tables A8 and A9 show estimates stratified by parental education and by level of area deprivation.

Parental education is taken from the 1971 census form, which has two questions on education, one question asking whether a respondent has the following qualifications: 1) GCE A-level or Higher School Certificate (HSC), 2) Higher grade of Scottish Certificate of Education (SCE) or Higher grade of Scottish Leaving Certificate (SLC), 3) Ordinary National Certificate (ONC) or Ordinary National Diploma (OND), or 4) None of these. The second question requests the respondent to indicate if they have any


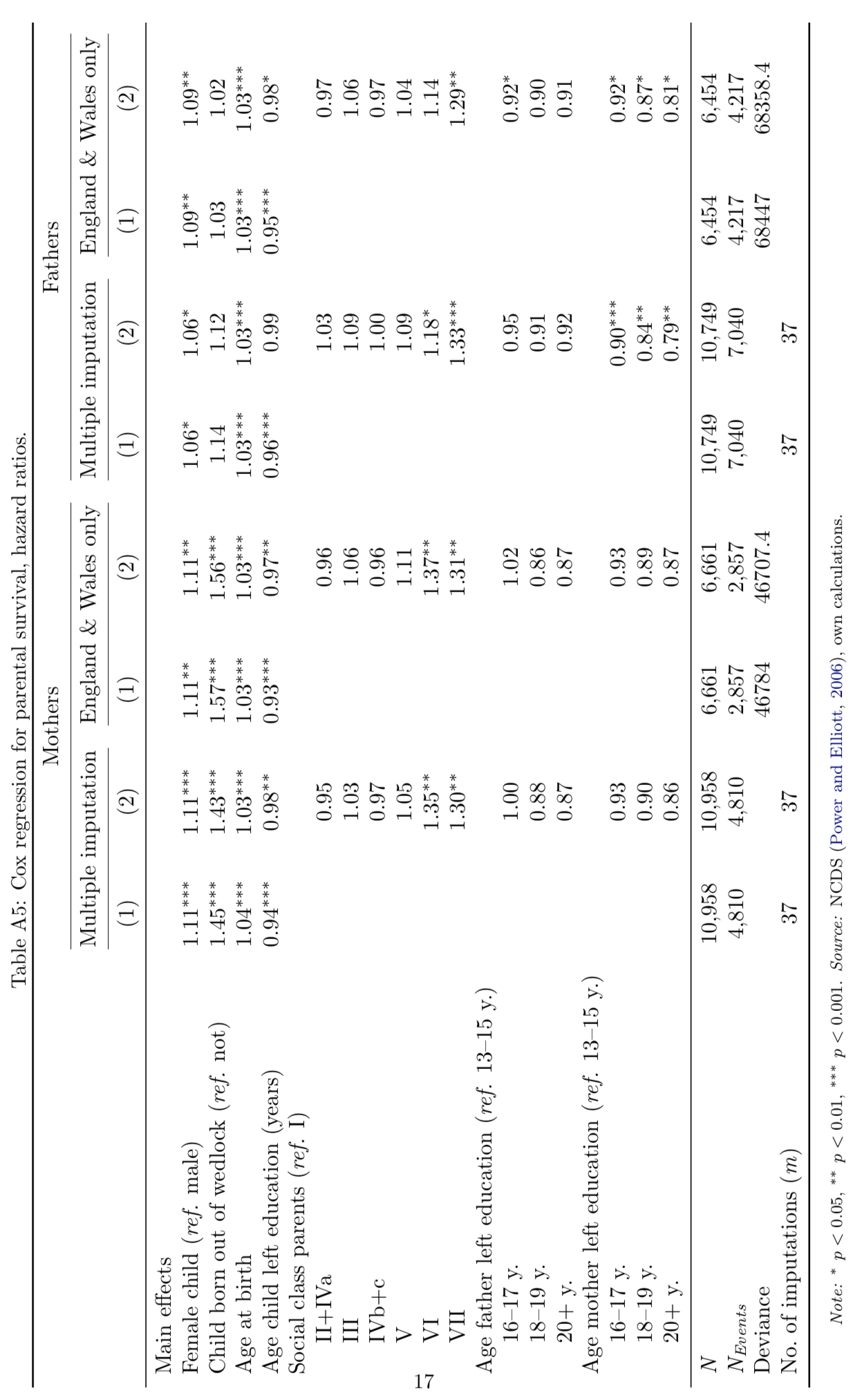


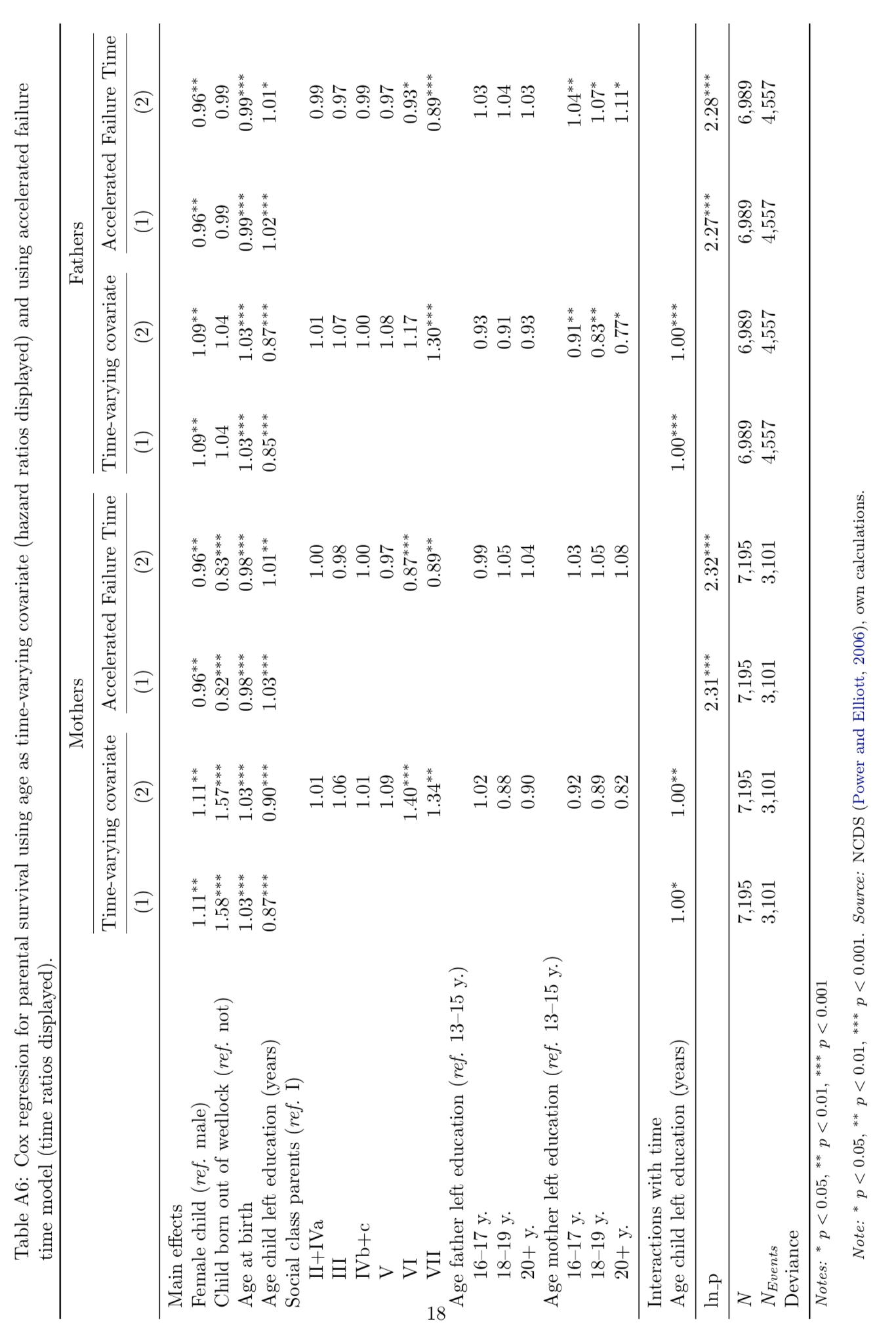


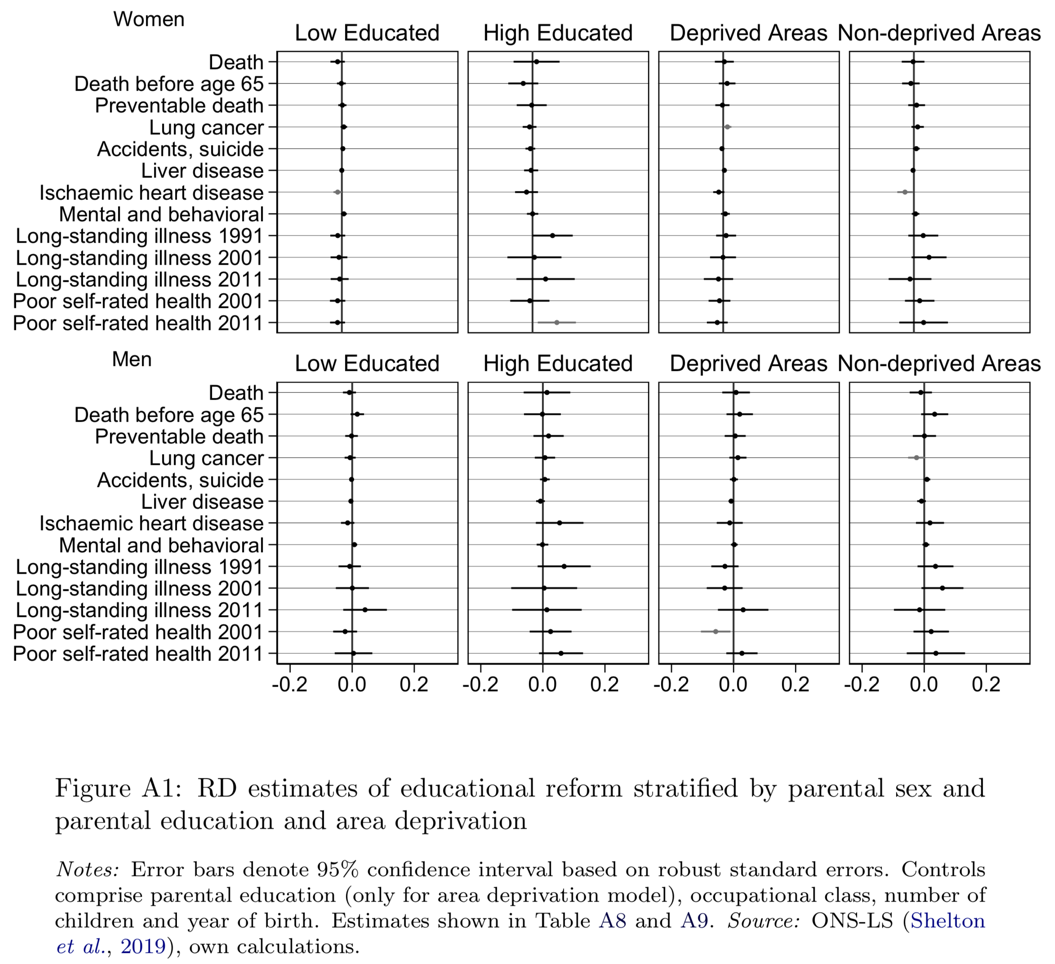


of the following qualifications: Higher National Certificate (HNC) or Higher National Diploma (HND), Nursing qualifications, Teaching qualifications, Degrees, Graduate or corporate membership of professional institutions, and Any other professional or vocational qualifications. For our analyses, we count affirmative responses to option 4) in the first question as being lower educated (which applies to ca. 90 per cent of the sample) and any other response as being higher educated.

In addition, we stratify the analyses by quintiles of the Carstairs index of deprivation (Morris and Carstairs, 1991), comparing the two lowest quintiles with the top-two quintiles (Norman and Boyle, 2014). The index is a well-established geographical measure of material deprivation and is based on four variables, namely regional unemployment, overcrowded households, households without cars, and households with a household head in a low occupational class. The Carstairs Index has previously been used in conjunction with the ONS-LS (Boyle *et al.*, 2004; Norman and Boyle, 2014; Norman *et al.*, 2005). The index is calculated at the ward level, which on average comprise 5,500 residents, and of which there are 7,707 in England and 881 in Wales. We measure the Carstairs index at the latest census for sample members who are alive at the end of the follow-up period, otherwise at the census closest to their time of death.

Among low-educated women and women in deprived areas, we do not find the same health benefits of more educated children that we observed for occupational class. A comparison of findings for occupational class with those for parental education and area deprivation, reveals that our findings are sensitive to the chosen measure of socio-economic position (Figures A1). Figure A1 contains an unexpected result. Children’s education reduces the risk of dying from ischaemic heart disease only for lower-educated women (-1*.*9%, -3*.*5%– -0*.*2%), but children’s education increases the risk of higher-educated women to report poor self-rated health in 2011 (9*.*1%, 1*.*8–16*.*5%).

The right-hand panels of Figure A1 show results by area deprivation, suggesting that children’s education reduces the risk of ischaemic heart disease (-3*.*4%, -6*.*4%– -0*.*5%) of women living in non-deprived areas (which is in line with the finding for occupational class but not education). For women in deprived areas children’s education increases the risk of dying from lung cancer (1*.*6%, 0*.*1%–3*.*2%). For men, stratifying by area deprivation yields two other findings: Educated children reduce the risk of men in deprived areas to report poor self-rated health in 2001 (-5*.*8%, -10*.*5%– -1*.*1%) and reduce the risk of dying from lung cancer in non-deprived areas (-2*.*5%, -5*.*2%– -0*.*2%).

**Number of children as stratifying variable** Table A7 presents models stratified by the number of children parents have. The rationale for this robustness check is that effects might be especially pronounced among parents with only one child affected by the reform rather than among parents with more children, some of whom will not be affected by the reform. This robustness check did not reveal any significant effects, which is in line with our overall findings.

*
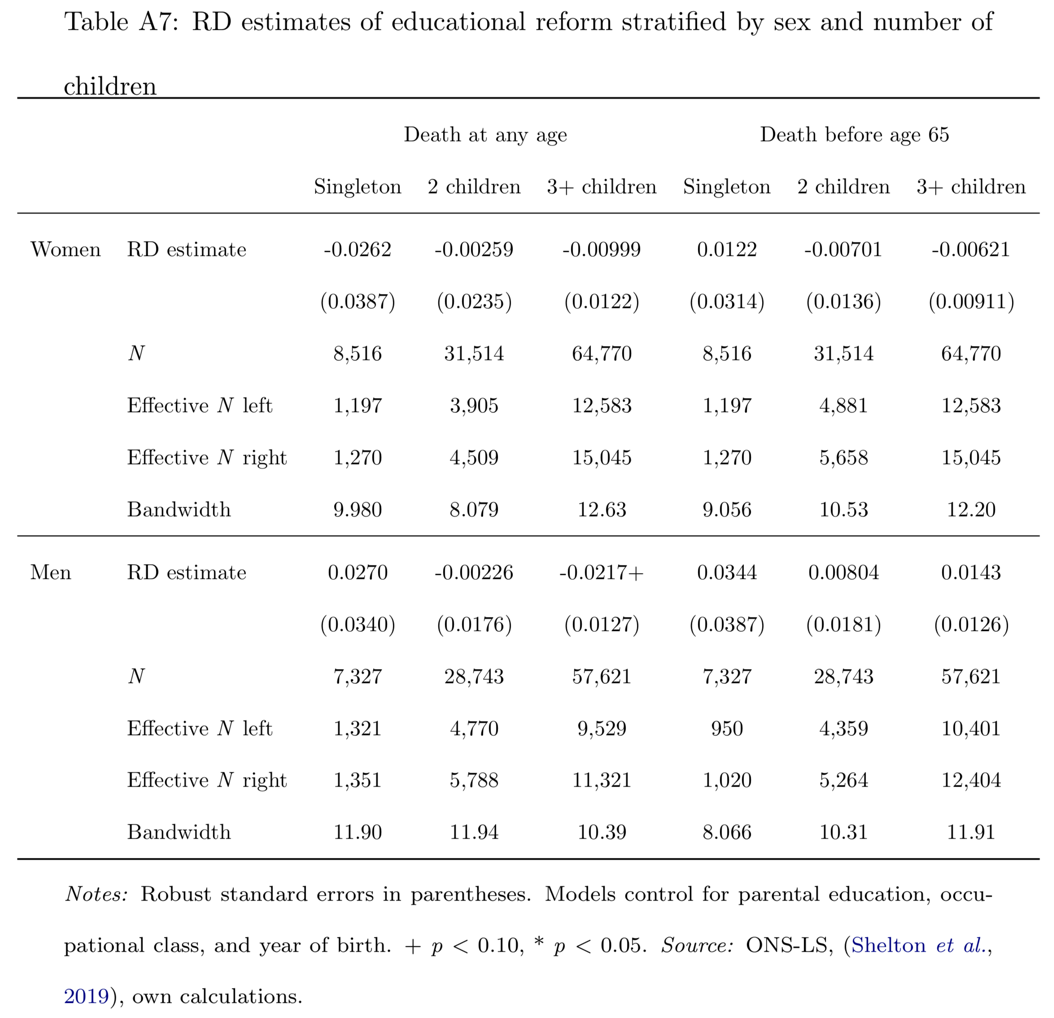
*

**Different bandwidths** Table A10 investigates the effects of choosing different bandwidths around the threshold date. The results remain the same when different bandwidths are selected, demonstrating low sensitivity to the bandwidth chosen.


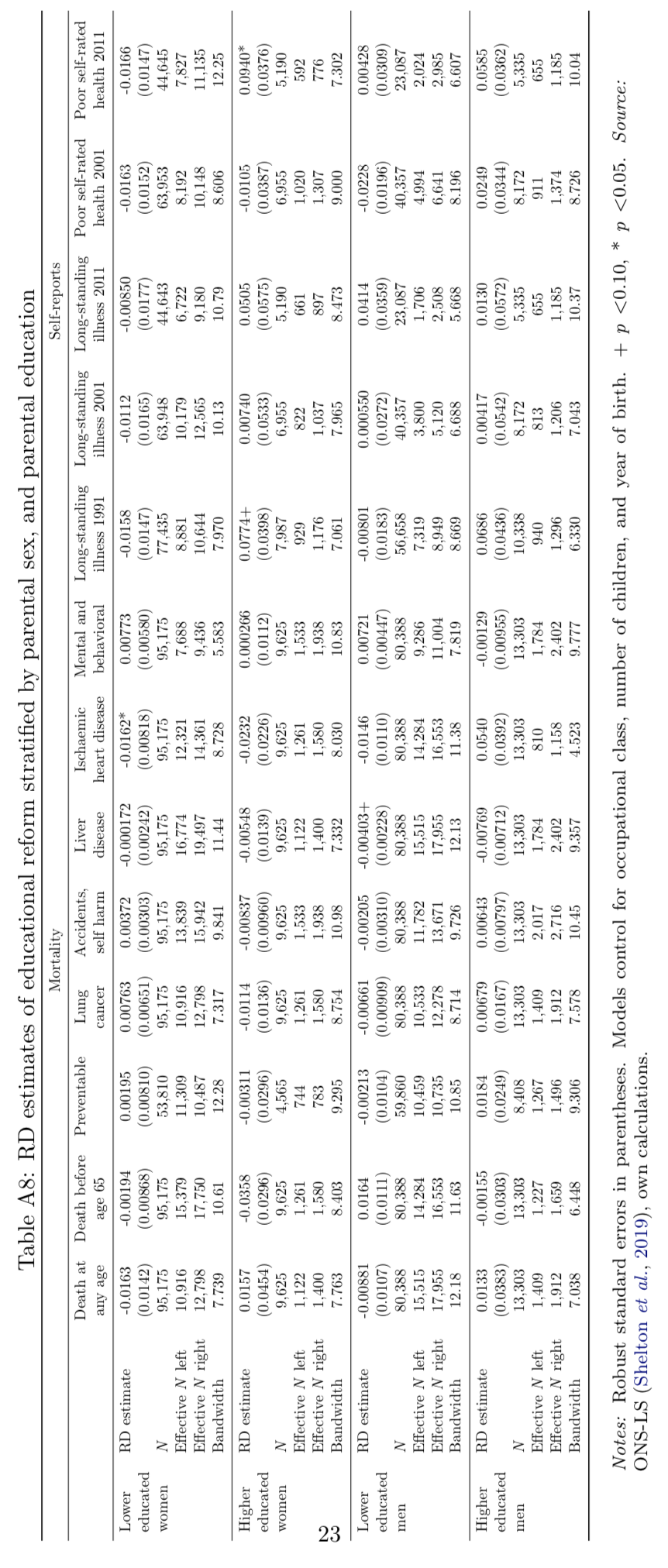


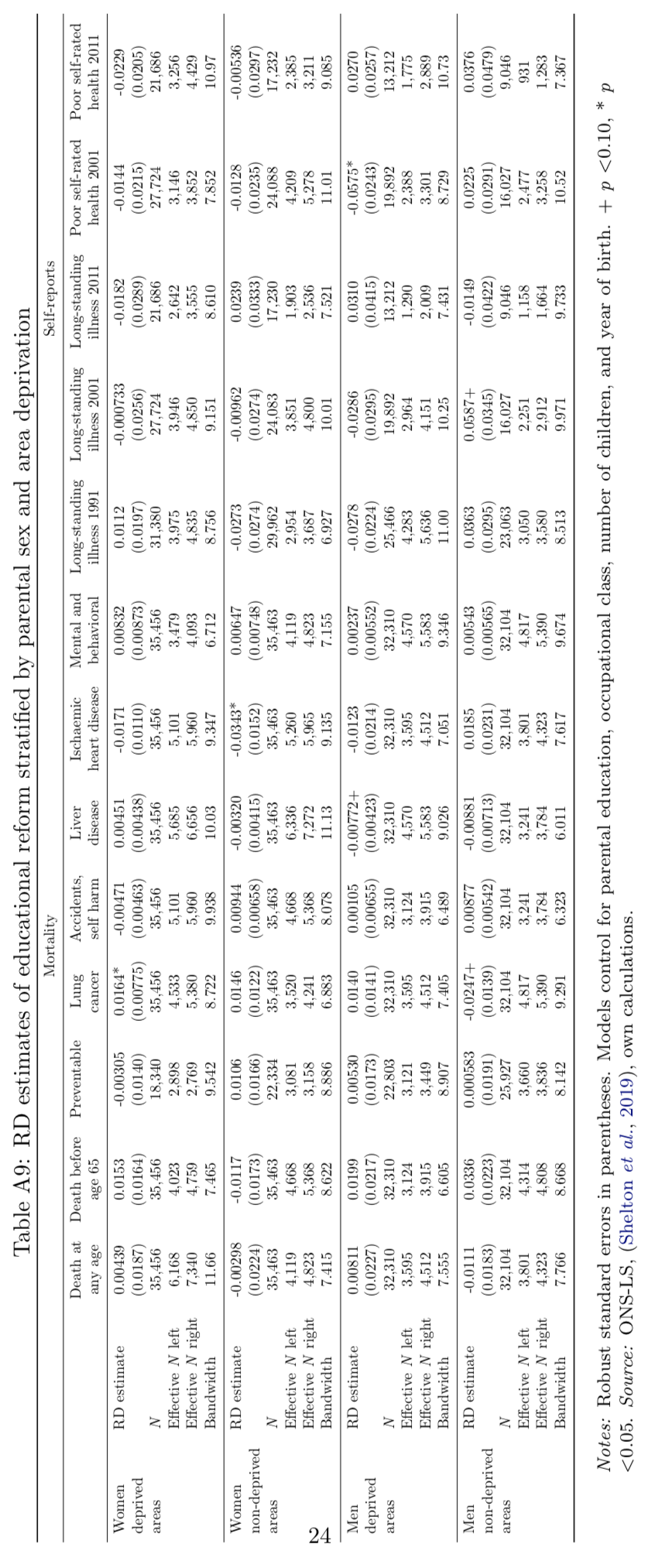


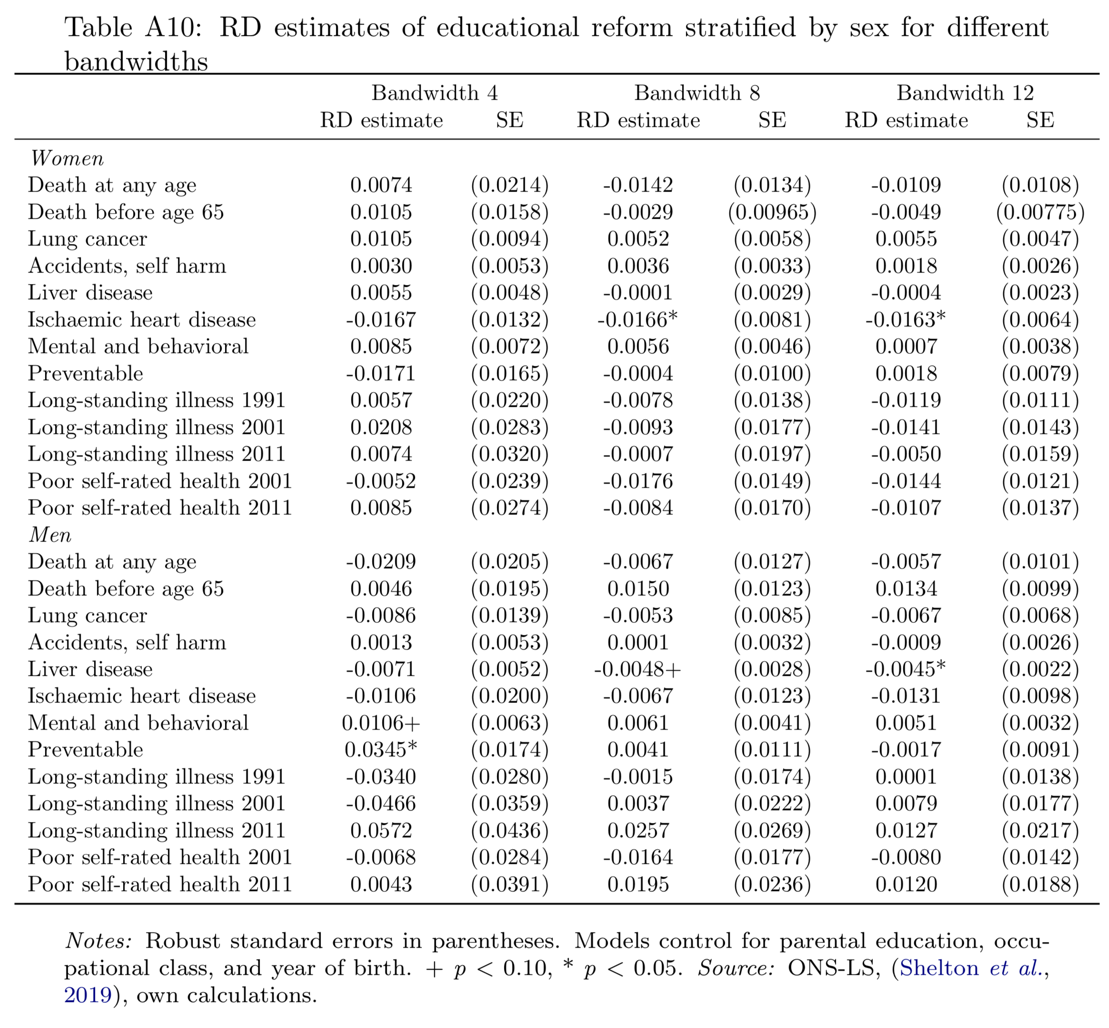


**Power Analysis** In order to check we have sufficient power for our analysis, we report the regression discontinuity power analysis implemented following Cattaneo, Titiunik, and Vazquez-Bare (2019). The estimated power analysis uses the optimal bandwidth, local parametric regression, bias-correction, and robust standard errors specified by Calonico, Cattaneo, and Titiunik (2014). Table A.11 provides the power estimates for T=1 which represents effects equal to half the standard deviation of the outcome for the untreated with a statistical significance level of 0.05. From Tables A.11 we observe that the power levels are all 1.00 across all of the outcomes for a medium-size effect. These results are greater than the common power threshold of 0.80. Moreover, we run additional analysis to examine the power even for smaller effect sizes. From Table A.12 and A.13 we observe that our analyses are able to detect smaller effects such as T=0.5 (0.25σ) and T=0.8 (0.4σ) with a high level of confidence. Only with T=0.2 (0.1σ) we observe that some outcome could slightly underpower but this represents extremely small effect. All the power analyses are implemented in Stata using the package “rdpower” (Cattaneo, Titiunik, and Vazquez-Bare, 2019).

Table A11: RDD power estimates with T=1 (i.e. effects equal to half the standard deviation of the outcome for the untreated).

| Tau=1 | Female | Male |
| --- | --- | --- |
| Death at any age | 1 | 1 |
| Death before age 65 | 1 | 1 |
| Lung cancer | 1 | 1 |
| Accident, self-harm | 1 | 1 |
| Liver disease | 1 | 1 |
| Ischaemic heart diseases | 1 | 1 |
| Mental and behavioural | 1 | 1 |
| Preventable | 1 | 1 |
| Long-standing illness 1991 | 1 | 1 |
| Long-standing illness 2001 | 1 | 1 |
| Long-standing illness 2011 | 1 | 1 |
| Poor self-rated health 2001 | 1 | 1 |
| Poor self-rated health 2011 | 1 | 1 |

*Source*: ONS-LS, (Shelton et al. 2019), own calculations

Table A12: RDD power estimates at varying effect sizes from rdpower package in Stata for Female.

|  | 40% of a Standard Deviation | 25% of a Standard Deviation | 10% of a Standard Deviation |
| --- | --- | --- | --- |
| Death at any age | 1 | 1 | 0.85 |
| Death before age 65 | 1 | 1 | 0.92 |
| Lung cancer | 1 | 1 | 0.77 |
| Accident, self-harm | 1 | 1 | 0.79 |
| Liver disease | 1 | 1 | 0.65 |
| Ischaemic heart diseases | 1 | 1 | 0.78 |
| Mental and behavioural | 1 | 1 | 0.74 |
| Preventable | 1 | 1 | 0.66 |
| Long-standing illness 1991 | 1 | 1 | 0.72 |
| Long-standing illness 2001 | 1 | 1 | 0.75 |
| Long-standing illness 2011 | 1 | 1 | 0.52 |
| Poor self-rated health 2001 | 1 | 1 | 0.63 |
| Poor self-rated health 2011 | 1 | 1 | 0.66 |

*Source*: ONS-LS, (Shelton et al. 2019), own calculations

Table A13: RDD power estimates at varying effect sizes from rdpower package in Stata for Male.

|  | 40% of a Standard Deviation | 25% of a Standard Deviation | 10% of a Standard Deviation |
| --- | --- | --- | --- |
| Death at any age | 1 | 1 | 0.83 |
| Death before age 65 | 1 | 1 | 0.87 |
| Lung cancer | 1 | 1 | 0.8 |
| Accident, self-harm | 1 | 1 | 0.85 |
| Liver disease | 1 | 1 | 0.77 |
| Ischaemic heart diseases | 1 | 1 | 0.83 |
| Mental and behavioural | 1 | 1 | 0.69 |
| Preventable | 1 | 1 | 0.71 |
| Long-standing illness 1991 | 1 | 1 | 0.59 |
| Long-standing illness 2001 | 1 | 1 | 0.49 |
| Long-standing illness 2011 | 1 | 0.89 | 0.25 |
| Poor self-rated health 2001 | 1 | 1 | 0.51 |
| Poor self-rated health 2011 | 1 | 0.89 | 0.25 |

*Source*: ONS-LS, (Shelton et al. 2019), own calculations

**References**

***NCDS (Power and Elliott, 2006) data sets used in empirical analysis 1***

Bukodi, Erzs´ebet, 2017. *National Child Development Study and 1970 British Cohort Study Educational Qualifications Histories, 1981–2009*. London: Center for Longitudinal Studies. [doi: 10.5255/UKDA-SN-8127-1.](about:blank)

Center for Longitudinal Studies, 2008a. *National Child Development Study. Sweep 6, 1999–2000*. London: Joint Center for Longitudinal Research, 2nd edition. [doi: 10.5255/UKDA-SN-5578-1.](about:blank)

——, 2008b. *National Child Development Study. Sweep 7, 2004–5*. Colchester:

UK Data Service, 3rd edition. [doi: 10.5255/UKDA-SN-5579-1.](about:blank)

——, 2012. *National Child Development Study. Sweep 8, 2008–9*. Colchester:

UK Data Service, 3rd edition. [doi: 10.5255/UKDA-SN-6137-2.](about:blank)

——, 2014. *National Child Development Study. Childhood Data, Sweeps 0–3,*

*1958–74*. London: National Birthday Trust Fund. [doi: 10.5255/UKDA-SN5565-2.](about:blank)

——, 2015. *National Child Development Study. Sweep 9, 2013*. London: UK Data Service. [doi: 10.5255/UKDA-SN-7669-1.](about:blank)

Power, Chris and Jane Elliott, 2006. ‘1958 British Birth Cohort (National Child Development Study).’ *International Journal of Epidemiology* 35(1): 34–41. [doi: 10.1093/ije/dyi183.](about:blank)

***HSE (Mindell*** *et al.****, 2012) data sets used in Figure 3***

Joint Health Surveys Unit of Social and Community Planning Research and

University College London, 2017a. *Health Survey for England 1994*. London: UK Data Service, 5th edition. [doi: 10.5255/UKDA-SN-3640-2.](about:blank)

——, 2017b. *Health Survey for England 1995*. London: UK Data Service, 5th edition. [doi: 10.5255/UKDA-SN-3796-2.](about:blank)

——, 2017c. *Health Survey for England 1996*. London: UK Data Service, 5th edition. [doi: 10.5255/UKDA-SN-3886-2.](about:blank)

——, 2017d. *Health Survey for England 1997*. London: UK Data Service, 4th edition. [doi: 10.5255/UKDA-SN-3979-2.](about:blank)

Mindell, Jennifer, Jane P. Biddulph, Vasant Hirani, Emanuel Stamatakis, Rachel Craig, Susan Nunn, and Nicola Shelton, 2012. ‘The Health Survey for England.’ *International Journal of Epidemiology* 41(6): 1585–1593. [doi:](about:blank)

[10.1093/ije/dyr199.](about:blank)

Natcen Social Research and Royal Free and University College Medical School,

Department of Epidemiology and Public Health, 2015. *Health Survey for England 2010*. London: UK Data Service, 3rd edition. [doi: 10.5255/UKDASN-6986-3.](about:blank)

Natcen Social Research and University College London, Department of Epidemiology and Public Health, 2014. *Health Survey for England 2012*. London: UK Data Service. [doi: 10.5255/UKDA-SN-7480-1.](about:blank)

——, 2015. *Health Survey for England 2013*. London: UK Data Service. [doi: 10.5255/UKDA-SN-7649-1.](about:blank)

National Center for Social Research and University College London, Department of Epidemiology and Public Health, 2010a. *Health Survey for England 1999*. London: UK Data Service, 4th edition. [doi: 10.5255/UKDA-SN-4365-1.](about:blank)

——, 2010b. *Health Survey for England 2001*. London: UK Data Service, 3rd edition. [doi: 10.5255/UKDA-SN-4628-1.](about:blank)

——, 2010c. *Health Survey for England 2004*. London: UK Data Service, 2nd edition. [doi: 10.5255/UKDA-SN-5439-1.](about:blank)

——, 2010d. *Health Survey for England 2007*. London: UK Data Service, 2nd edition. [doi: 10.5255/UKDA-SN-6112-1.](about:blank)

——, 2011. *Health Survey for England 2006*. London: UK Data Service, 4th edition. [doi: 10.5255/UKDA-SN-5809-1.](about:blank)

——, 2013. *Health Survey for England 2008*. London: UK Data Service, 4th edition. [doi: 10.5255/UKDA-SN-6397-2.](about:blank)

Office of Population Censuses and Surveys, Social Survey Division, 1997a. *Health*

*Survey for England 1991–2. Combined Data File*. London: UK Data Service, 2nd edition. [doi: 10.5255/UKDA-SN-3238-1.](about:blank)

——, 1997b. *Health Survey for England 1993*. London: UK Data Service, 2nd edition. [doi: 10.5255/UKDA-SN-3316-1.](about:blank)

University College London, Department of Epidemiology and Public Health and

National Center for Social Research, 2010a. *Health Survey for England 1998*. London: UK Data Service, 5th edition. [doi: 10.5255/UKDA-SN-4150-1.](about:blank)

——, 2010b. *Health Survey for England 2000*. London: UK Data Service, 4th edition. [doi: 10.5255/UKDA-SN-4487-1.](about:blank)

——, 2010c. *Health Survey for England 2002*. London: UK Data Service, 2nd edition. [doi: 10.5255/UKDA-SN-4912-1.](about:blank)

——, 2010d. *Health Survey for England 2003*. London: UK Data Service, 2nd edition. [doi: 10.5255/UKDA-SN-5098-1.](about:blank)

——, 2011. *Health Survey for England 2005*. London: UK Data Service, 3rd edition. [doi: 10.5255/UKDA-SN-5675-1.](about:blank)

——, 2013. *Health Survey for England 2011*. London: UK Data Service. [doi: 10.5255/UKDA-SN-7260-1.](about:blank)

——, 2015. *Health Survey for England 2009*. London: UK Data Service, 3rd edition. [doi: 10.5255/UKDA-SN-6732-2http://dx.doi.org/10.5255/UKDA-SN-6732-2](about:blank)

***List of studies included in Figure 1***

De Neve, Jan-Walter and Gu¨nther Fink, 2018. ‘Children’s Education and Parental Old Age Survival. Quasi-Experimental Evidence on the Intergenerational Effects of Human Capital Investment.’ *Journal of Health Economics* 58: 76–89. [doi: 10.1016/j.jhealeco.2018.01.008.](about:blank)

De Neve, Jan-Walter and Guy Harling, 2017. ‘Offspring Schooling Associated with Increased Parental Survival in Rural KwaZulu-Natal, South Africa.’  *Social Science and Medicine* 176: 149–157. [doi:](about:blank) [10.1016/j.socscimed.2017.01.015.](about:blank)

Elo, Irma T., Pekka Martikainen, and Mikko Aaltonen, 2018. ‘Children’s Educational Attainment, Occupation, and Income and Their Parents’ Mortality.’ *Population Studies* 72(1): 53–73. [doi: 10.1080/00324728.2017.1367413.](about:blank)

Friedman, Esther M. and Robert D. Mare, 2014. ‘The Schooling of Offspring and the Survival of Parents.’ *Demography* 51(4): 1271–1293. [doi: 10.1007/s13524014-0303-z.](about:blank)

Jiang, Nan, 2019. ‘Adult Children’s Education and Later-Life Health of Parents in China. The Intergenerational Effects of Human Capital Investment.’ *Social Indicators Research* 145: 257–278. [doi: 10.1007/s11205-019-02109-9.](about:blank)

Lee, Chioun, 2018. ‘Adult Children’s Education and Physiological Dysregulation Among Older Parents.’ *Journals of Gerontology B* 73(6): 1143–1154. [doi: 10.1093/geronb/gbx044.](about:blank)

Lee, Chioun, Dana A. Glei, Noreen Goldman, and Maxine Weinstein, 2017. ‘Children’s Education and Parents’ Trajectories of Depressive Symptoms.’ *Journal of Health and Social Behavior* 58(1): 86–101. [doi:](about:blank) [10.1177/0022146517690200.](about:blank)

Lundborg, Petter and Kaveh Majlesi, 2018. ‘Intergenerational Transmission of

Human Capital. Is It a One-Way Street?’ *Journal of Health Economics* 57: 206–220. [doi: 10.1016/j.jhealeco.2017.12.001.](about:blank)

Ma, Mingming, 2019. ‘Does Children’s Education Matter for Parents’ Health and Cognition? Evidence from China.’ *Journal of Health Economics* 66: 222–240. [doi: 10.1016/j.jhealeco.2019.06.004.](about:blank)

Sabater, Albert and Elspeth Graham, 2016a. ‘Intergenerational Exchanges, Children’s Education, and Parents’ Longevity in Europe.’ *ESRC Centre for Population Change Working Paper* 77.

——, 2016b. ‘The Role of Children’s Education for the Mental Health of Aging Migrants in Europe.’ *GeroPsych* 29(2): 81–92. [doi: 10.1024/16629647/a000145.](about:blank)

Torssander, Jenny, 2013. ‘From Child to Parent? The Significance of Children’s Education for Their Parents’ Longevity.’ *Demography* 50(2): 637–659. [doi: 10.1007/s13524-012-0155-3.](about:blank)

——, 2014. ‘Adult Children’s Socioeconomic Positions and Their Parents’ Mortality. A Comparison of Education, Occupational Class, and Income.’ *Social Science and Medicine* 122: 148–156. [doi: 10.1016/j.socscimed.2014.10.043.](about:blank)

Wolfe, Joseph D., Shawn Bauldry, Melissa A. Hardy, and Eliza K. Pavalko, 2018a. ‘Multigenerational Attainments, Race, and Mortality Risk among Silent Generation Women.’ *Journal of Health and Social Behavior* 59(3): 335–351. [doi: 10.1177/0022146518784596.](about:blank)

——, 2018b. ‘Multigenerational Socioeconomic Attainments and Mortality among Older Men. An Adjacent Generations Approach.’ *Demographic Research* 39(26): 719–752. [doi: 10.4054/DemRes.2018.39.26.](about:blank)

Yahirun, Jenjira J., Connor M. Sheehan, and Mark D. Hayward, 2016. ‘Adult Children’s Education and Parents’ Functional Limitations in Mexico.’ *Research on Aging* 38(3): 322–345. [doi: 10.1177/0164027515620240.](about:blank)

——, 2017. ‘Adult Children’s Education and Changes to Parents’ Physical Health in Mexico.’ *Social Science and Medicine* 181: 93–101. [doi:](about:blank)

[10.1016/j.socscimed.2017.03.034.](about:blank)

Yahirun, Jenjira J., Connor M. Sheehan, and Krysia N. Mossakowski, 2020a.‘Depression in Later Life. The Role of Adult Children’s College Education for Older Parents’ Mental Health in the United States.’ *Journals of Gerontology B* 75(2): 389–402. [doi: 10.1093/geronb/gby135.](about:blank) 10.1093/geronb/gby135.

Yahirun, Jenjira J., Sindhu Vasireddy, and Mark D. Hayward, 2020b. ‘The Education of Multiple Family Members and the Life Course Pathways to Cognitive Impairment.’ *Journals of Gerontology B* [doi: 10.1093/geronb/gbaa039.](about:blank)

Yang, Lei, Pekka Martikainen, and Karri Silventoinen, 2016. ‘Effects of Individual, Spousal, and Offspring Socioeconomic Status on Mortality Among Elderly People in China.’ *Journal of Epidemiology* 26(11): 602–609. [doi: 10.2188/jea.JE20150252.](about:blank)

Zimmer, Zachary, Heidi A. Hanson, and Ken R. Smith, 2016. ‘Offspring Socioeconomic Status and Parent Mortality Within a Historical Population.’ *Demography* 53(5): 1583–1603. [doi: 10.1007/s13524-016-0502-x.](about:blank)

Zimmer, Zachary, Albert I. Hermalin, and Hui-Sheng Lin, 2002. ‘Whose Education Counts? The Added Impact of Adult-Child Education on Physical Functioning of Older Taiwanese.’ *Journals of Gerontology B* 57(1): S23–S32. [doi: 10.1093/geronb/57.1.S23.](about:blank)

Zimmer, Zachary, Linda G. Martin, Mary Beth Ofstedal, and Yi-Li Chuang, 2007. ‘Education of Adult Children and Mortality of their Elderly Parents in Taiwan.’ *Demography* 44(2): 289–305. [doi: 10.1353/dem.2007.0020.](about:blank)
